# A suite of fluorescent timers for visualizing protein spatiotemporal dynamics in plants

**DOI:** 10.1126/sciadv.aef3538

**Published:** 2026-09-16

**Authors:** Yukihiro Nagashima, Xin Qiao, Suji Ye, Qiaochu Shen, Jun Liu, Delui Liu, Fausto Andrés Ortiz-Morea, Xue Ding, Vinita Sharma, Juan Dong, Libo Shan, Hisashi Koiwa

**Affiliations:** ^1^ Vegetable and Fruit Improvement Center and Department of Horticultural Sciences, Texas A&M University, College Station, TX 77843, USA.; ^2^ Waksman Institute of Microbiology, Rutgers, The State University of New Jersey, Piscataway, NJ 08854, USA.; ^3^ Department of Biochemistry and Biophysics, Texas A&M University, College Station, TX 77843, USA.; ^4^ Department of Molecular, Cellular, and Developmental Biology, University of Michigan, Ann Arbor, MI 48109, USA.; ^5^ Molecular and Environmental Plant Sciences, Texas A&M University, College Station, TX 77843, USA.

## Abstract

Fluorescent timers (FTs), including tandem FTs (tdFTs), enable real-time visualization of precise protein localization and turnover within living cells, an essential property for elucidating protein function. In plants, application of FT has been limited largely because of the mismatch between FT maturation kinetics and the slow growth rate of plant cells. Here, we systematically evaluated multiple FT configurations to identify tdFTs optimized for plant systems. Using *Arabidopsis thaliana*, we tested a range of fluorescent proteins (FPs) differing in maturation times. Functional tdFT expression was confirmed in diverse organelles. Selected tdFT configurations were developed to track protein biogenesis/turnover in autoimmunity, protein transport in abiotic stress, and polarized protein translocation in determination of cell fate. When the large size of tdFT interfered with protein mobility, we developed a bipartite FT, separately tagging proteins with different FPs. This resource provides a framework for selecting FTs to dissect dynamic protein behaviors in plant cells.

## INTRODUCTION

Spatiotemporal distributions of nascent and aged proteins are an integral part of cellular signaling. “Life of protein” is regulated at multiple levels starting from protein production to modification, activation, transport, recycle, and degradation within the cell. Moreover, these regulations are modulated by endogenous and environmental cues. The juvenile and aged proteins produced from the same gene can execute distinct functions, yet current technology is limited in monitoring protein ages in plant cells. Fluorescent timer (FT) proteins are molecular tools capable of visualizing in vivo spatiotemporal protein distributions. The first FT reported was a DsRED-E5, a fluorescent protein changing colors from green to red over time, which was used to report gene expression dynamics (*1*). Later, several monomeric FTs (mFTs) based on mCherry, mRuby, and mKusabira-Orange, which are more suitable for tagging individual protein than tetrameric DsRED-E5, had been reported (*2*, *3*). Still, mFT proteins have many limitations including relatively weak fluorescence levels, a limited choice in maturation kinetics, and tendency to show excitation light–induced chromophore maturation (*3*).

An innovation, tandem FT (tFT/tdFT), overcame several problems associated with mFTs by tandemly linking two superior fluorescent proteins, typically monomeric green fluorescent protein (GFP) and red fluorescent protein (RFP) to a protein of interest (POI). In original tdFT, a fast-maturing monomeric superfolder GFP (msfGFP; maturation time: *t*_0.5_ = 13.6 min) was used to detect total POI levels quickly after translation, and mCherry with a slower maturation time [*t*_0.5_ = ∼40 min (*4*)] was used to monitor age-dependent signals. Both proteins are brighter than mFTs at maturity, making the assay more sensitive. The age of POI at a specific location is determined on the basis of the ratio of RFP to GFP in vivo in real time. Successful application of tdFT in yeast and animal systems is increasing (*5*–*12*); however, only a handful of cases have deployed tdFTs to address fundamental questions in plant sciences (*13*, *14*). A wide application of the tdFT system is hampered by the lack of reporters with resolutions adequately characterized and optimized for plant systems, the complexity of data processing, and the lack of infrastructures to share the latest materials and knowledge for tdFT imaging.

One of the difficulties in using FT in plants is the slow growth rate of plants. Considering the doubling time of ∼90 min in yeast versus 31 to 35 hours in plants, plant proteins likely have longer turnover rates. The time range of tdFTs is determined by maturation kinetics of slow-maturing RFP; hence, rational selection of RFP with an adequate maturation time for the biological time frame of the chosen process is the key to tailor tdFTs for specific purposes. Now, an overwhelming number of RFPs (>170) have been reported. However, essential parameters were determined predominantly at 37°C both in vitro and in vivo. In plants with growth temperatures of 20° to 30°C, GFP/RFP maturation and turnover kinetics are expected to be substantially different. Moreover, plant cells have unique organelles with distinct pH environments, further influencing fluorescent protein behavior. To date, only mCherry- and monomeric Strawberry [mStrawberry (mSt)]–based tdFTs [tdFT(mCh) and tdFT(mSt), respectively] have been adapted in plants (*13*, *14*), which are far from a sufficient scale for providing a suite of tdFT designs suitable for different classes of proteins.

To develop a suite of FT molecules suitable for in diverse applications in plant biology, we systematically designed and characterized a series of FT configurations using the tdFT platform. Timer configurations were chosen on the basis of high brightness and maturation kinetics matching the time scale of different plant biological/cellular processes. The performance of FTs was recorded using both *Arabidopsis* protoplast transient expression and stable transgenic plants, providing valuable reference points for designing timer experiments. Also addressed were expected pitfalls of FT analyses, including the suitability of targeting FTs to intracellular organelles with different luminal pH levels and steric hindrance caused by a relatively large tdFT-tag. The latter was addressed by a bipartite configuration of timer proteins [bipartite FTs (bpFTs)], which minimizes steric hindrance and allows timer data to be directly comparable to the conventional fluorescent reporter data. The results collectively showed unexpected differences between predicted and in vivo–observed fluorescent signal kinetics of fluorescent proteins in planta. The systematic and empirical data collected in this study are an essential resource for effectively designing FT experiments for new biological systems in the plant community.

## RESULTS

### Rational design of tdFTs for plants

Adapting FTs for plants requires balancing cell growth rate, temperature, and fluorophore maturation kinetics. Compared to yeast, plant cells grow and divide more slowly, suggesting that a slower-maturing RFP may better capture protein ages (*7*). However, the lower growth temperatures for plants further slow fluorophore maturation (*15*), which affects the detection efficiency and necessitates empirical optimization of each timer’s measurable range. To define this range, we compared maturation kinetics of a set of tdFTs designed from published RFP parameters. Our previously characterized tdFT(mSt) fused to the membrane protein KORRIGAN1 (KOR1), which is the only tdFT with time course information in plants, provided a baseline reference (*14*). GFP signals appeared within 1-hour postinduction of tdFT expression, while the mSt signal emerged after ∼4 hours and continued increasing for 10 to 12 hours (*14*). This timescale corresponded well with the typical duration of protein trafficking in the plant secretory pathway, indicating that mSt (*t*_0.5_ at 37°C = 50 min) is suitable for monitoring protein biogenesis. By contrast, slower-maturing RFPs may better track protein degradation or cell lineage.

**Fig. 1.**
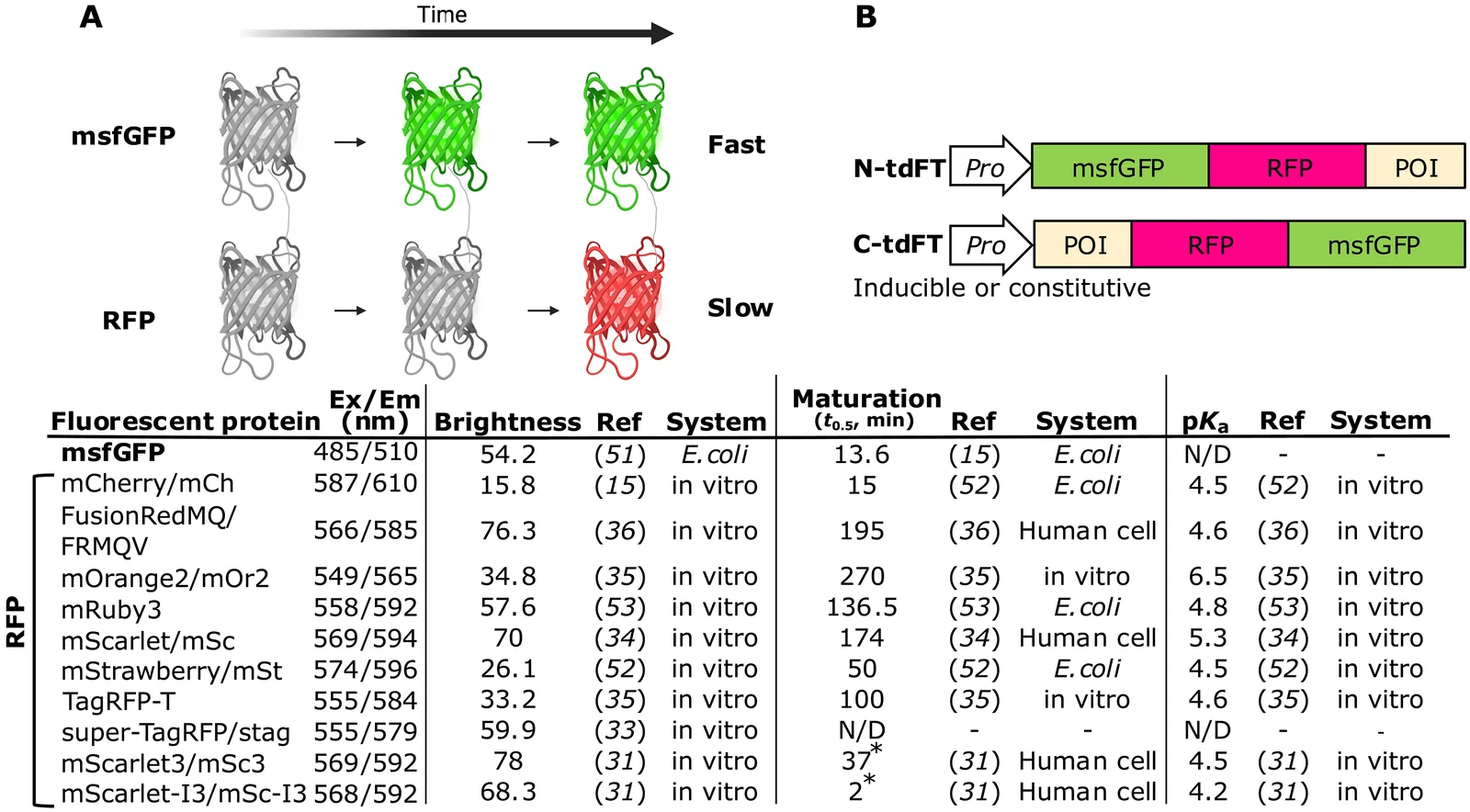
tdFTs for plants. (**A**) Mechanism of the tdFT reporter protein. Maturation of GFP (msfGFP) is fast, while that of RFP is slow. The table underneath shows a series of RFPs with different maturation half-life times (*t*_0.5_; obtained from www.fpbase.org) tested in this study. *Relative to mTurquoise2. N/D indicates no data. Created in BioRender. Y. Nagashima (2026), https://biorender.com/l30k142. (**B**) Schematics show the N-tdFT and C-tdFT with POI. *Pro*, promoter. For constitutive expression, an enhanced CaMV 35*S* promoter was used for transient assays in Fig. 2, while for inducible expression, the β-estradiol–inducible promoter was used for assays in Fig. 5. Basic parameters of GFP and RFP used in this study were summarized at the bottom (*15*, *31*, *33*–*36*, *51*–*53*).

RFP brightness is another critical determinant of tdFT performance as brighter RFPs generate detectable signals early, expanding the measurable window and the dynamic range. On the basis of these rationales, we selected seven bright RFPs spanning a range of maturation rates—FusionRedMQV (FRMQV), monomeric Orange2 [mOrange2 (mOr2)], monomeric Ruby3 (mRuby3), monomeric Scarlet [mScarlet (mSc)], TagRFP-T, and super-tagRFP (stag)—with mSt included as a reference (Fig. 1A). Each RFP was fused with msfGFP in two configurations, N-terminal tdFT (N-tdFT) and C-terminal tdFT (C-tdFT) (Fig. 1B), to assess potential proteolytic sensitivity from either terminus (*16*). The base constructs were generated in Gateway-compatible *pEnEOi* vectors driven by the *EL2* promoter, enabling constitutive expression in plant cells (*17*).

### Evaluating kinetics and subcellular targeting of tdFTs in *Arabidopsis* protoplasts

We next evaluated properties and maturation kinetics of tdFTs expressed in *Arabidopsis* leaf mesophyll protoplasts. Expression of tdFTs and transformation efficiency were verified using fluorescence microscopy (fig. S1, A and B). For this test, we used a C-tdFT configuration to ensure that the translation of RFP occurs before the translation of the GFP domain. In this configuration, the presence of GFP signals ensures the successful translation of the full-length tdFT. Notably, GC contents of tdFT coding sequences used in this study (55 to 62%) are substantially higher than the typical *Arabidopsis* coding sequence (44%). However, we observed that different tdFTs produced comparable GFP fluorescence, the indicator of overall expression levels. These observations are consistent with a recent report showing that transgenes with high GC content and unoptimized codon usage express better than codon-optimized transgenes in plants (*18*). On the other hand, RFP/GFP ratios at 12 hours posttransfection across different tdFTs were variable under the unified condition (fig. S1C), indicating their distinct maturation behaviors.

**Fig. 2.**
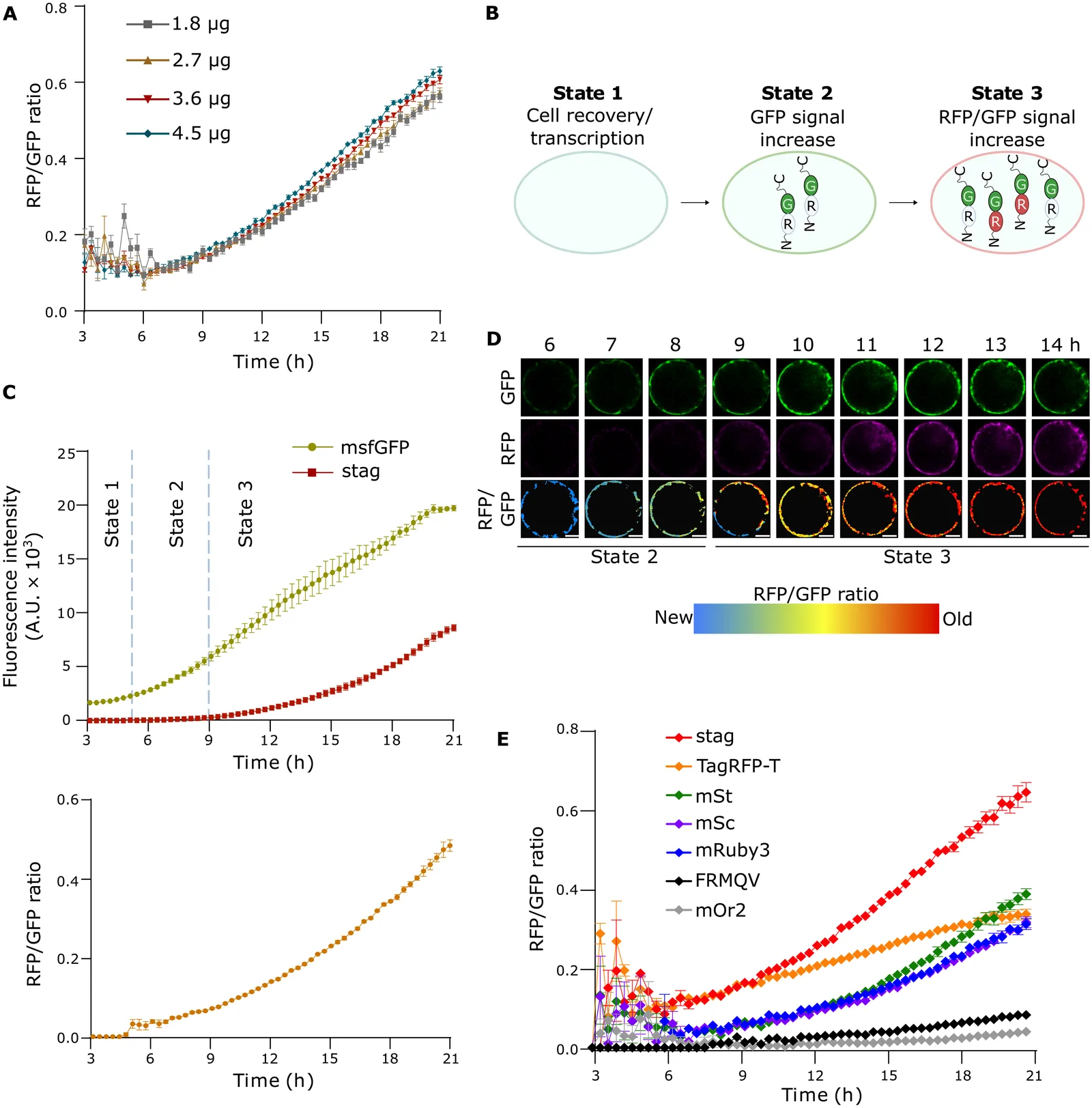
Application of tdFTs for dynamic analysis in *Arabidopsis* protoplasts. (**A**) The RFP/GFP ratio of tdFT(stag) is independent of DNA dosage exhibiting similar RFP/GFP ratios in response to a series of DNA amount used for protoplast transfection. h, hours. (**B**) Diagram illustrating three states of cells showing different fluorescent signal patterns based on the maturation time of GFP and RFP proteins. Green, white, and red circles indicate mature GFP, nascent RFP, and mature RFP, respectively. (**C**) Quantification of tdFT fluorescence. The top panel shows the fluorescence intensity of stagRFP and msfGFP at the indicated time points, with the expected state in (B). The lower panel shows the RFP and GFP ratio. See also fig. S1. A.U., arbitrary units. (**D**) Time course of RFP and GFP fluorescence signals from *Arabidopsis* protoplasts expressing tdFT(stag). Representative protoplast images corresponding to the quantification data in (C) were shown 6 to 14 hours after transfection with states 2 and 3 underlined. (**E**) Comparison of tdFT with different RFP variants in the *Arabidopsis* protoplasts. The optimal combination for the tdFT was identified as stagRFP and msfGFP. *n* = 3. Error bars represent the standard error of the mean (SEM).

To monitor tdFT kinetics in real time, we established a microtiter plate–based fluorescence detection system, enabling continuous measurement of tdFT signals in living cells. Calibration with varying plasmid DNA concentrations confirmed that while transformation efficiency increased with DNA amount, the RFP/GFP ratio, reflecting tdFT maturation, remained unaffected (Fig. 2A), demonstrating the tolerance of tdFT readout to the variability of transformation efficiency. Time-course analyses revealed a characteristic three-state maturation sequence (Fig. 2B). During the initial ∼5 hours (state 1), weak autofluorescence likely arising from transfection stress was observed (Fig. 2, B and C). This was followed by a GFP-only phase (state 2) and subsequent emergence of RFP fluorescence (state 3; Fig. 2, B to D), consistent with the established three-state maturation model for FTs (*16*).

We then compared tdFTs side by side from 3 to 21 hours posttransfection (Fig. 2E). On the basis of maturation kinetics and dynamic range, tdFTs were grouped into three classes. tdFT(stag) displayed the highest ratiometric dynamic range among all tested tdFT configurations, having a short state 1 period without signals and expanded states 2 and 3 that continuously increase the RFP/GFP ratio from 4 to 21 hours posttransfection (class 1a; Fig. 2E). tdFT(TagRFP-T) showed an initial signal profile similar to tdFT(stag); however, the RFP/GFP ratio did not increase as quickly as that of tdFT(stag) subsequently. At 20 hours after transfection, the RFP/GFP ratio of tdFT(TagRFP-T) was similar to that of class 2 tdFTs (see below). We therefore designated tdFT(TagRFP-T) as class 1b. tdFTs with mSt, mSc, and mRuby3 produced a similar ratiometric curve, with tdFT(mSt) slightly above the other two. RFP signals became detectable at 9 to 12 hours posttransfection and continued to develop (class 2; Fig. 2E). tdFT(FRMQV) and tdFT(mOr2) with maturation times of 195 and 270 min exhibited limited ratiometric dynamic ranges and substantially slower maturation kinetics compared to other tdFTs. These tdFTs may be useful for long-term observations of slow processes but are not suitable for relatively fast cellular processes and observation of propagating cells (class 3). These results demonstrate that tdFTs function robustly in plant protoplasts and identify tdFT(stag) as a promising candidate for further characterizations of dynamic protein tracking, followed by TagRFP-T, mSt, mSc, and mRuby3.

**Fig. 3.**
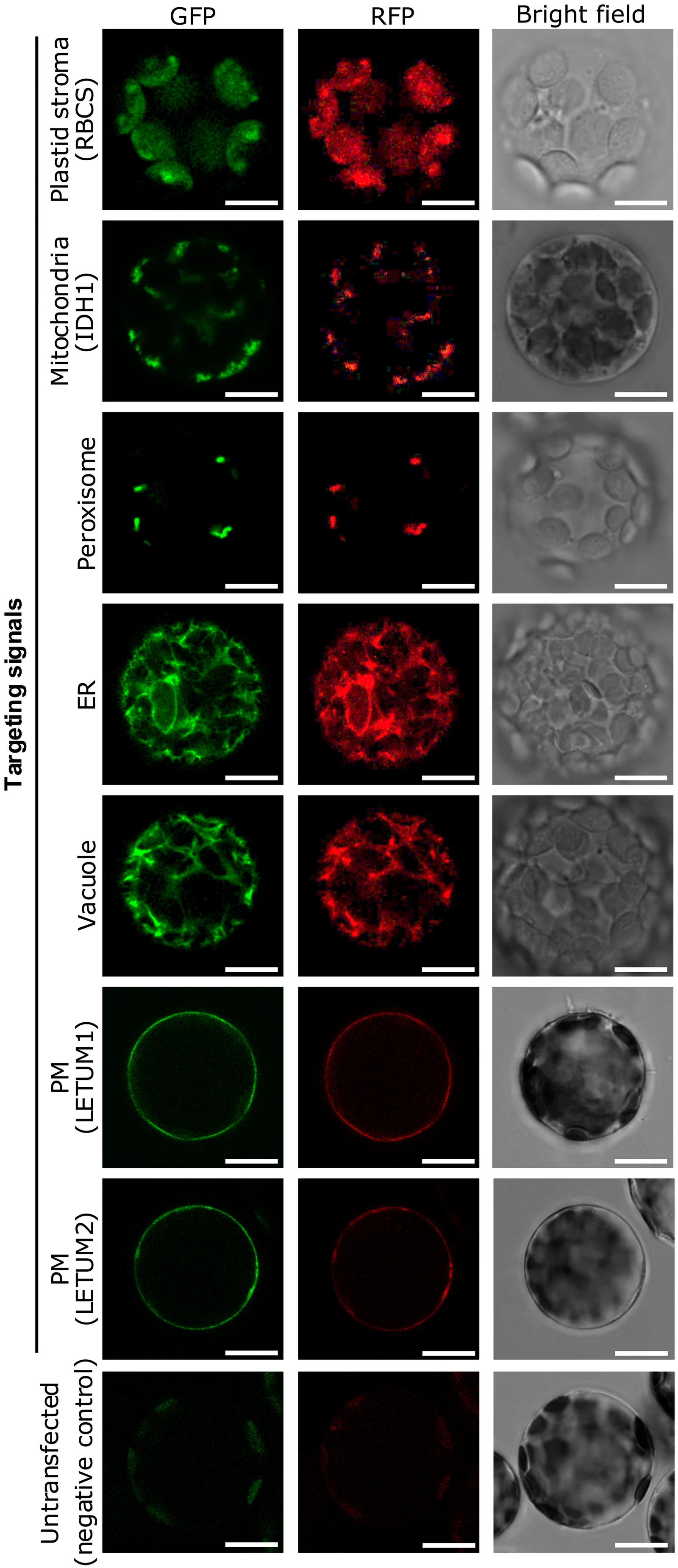
Targeting of tdFT(stag) to specific organelles with distinct luminal pH. tdFT(stag) fused to various subcellular targeting sequences was expressed in *Arabidopsis* protoplasts and detected by confocal laser microscopy. The targeted compartments included the plastid stroma (pH 7.2), mitochondria (pH 8.1), peroxisome (pH 8.4), and ER (pH 7.1 to 7.3). The expression of tdFT-tagged proteins remained stable and unaffected by the differing pH condition within each organelle. A protoplast with the vacuolar targeting signal showed an ER-like pattern. Protoplasts were prepared from 4-week-old *Arabidopsis* plants. Images were taken at 12 hours after transfection. RBCS, ribulose bisphosphate carboxylase/oxidase small subunit. Scale bars indicate 10 μm.

Because of the dimeric nature, tdFTs are larger than conventional fluorescent reporter proteins, thereby potentially interfering with organelle targeting efficiency. In addition, compartmentalized organelles often have different physiological parameters, for instance, iron, pH values, and redox status, likely influencing the fluorophore maturation. To test the subcellular versatility, tdFT(stag) was expressed with organelle-specific targeting signals, including chloroplasts, mitochondria, peroxisomes, endoplasmic reticulum (ER), plasma membrane (PM), and vacuoles. As shown in Fig. 3, fluorescence signals were successfully observed in chloroplasts, peroxisomes, ER, and PM using *Arabidopsis* protoplasts. Mitochondrial targeting was inefficient using targeting peptides from the *Arabidopsis* F_1_F_0_ adenosine triphosphatase subunit or *Saccharomyces cerevisiae* cytochrome C oxidase subunit COX4 (*19*) but succeeded with *Arabidopsis* full-length mitochondrially localized NAD^+^-dependent isocitrate dehydrogenase regulatory subunit IDH1–based targeting (*At*IDH1-tdFT), producing a fluorescence profile and localization comparable to *At*IDH1-GFP (*20*). However, when fused with a targeting signal for the vacuole using an N-terminal signal peptide and C-terminal AFVY motif (*21*), tdFT(stag) exhibited an ER-like localization pattern, suggesting failure to exit the ER (Fig. 3). Thus, current tdFT configurations are compatible with most organelles but not yet suitable for the lumen of post-ER compartments. By contrast, tdFT fused to the cytoplasmic domain of PM proteins LETUM1/2 (LET1/2) was successfully delivered to the PM (Fig. 3).

### Application of a protoplast-based tdFT system for the analysis of PM receptor kinase turnover

**Fig. 4.**
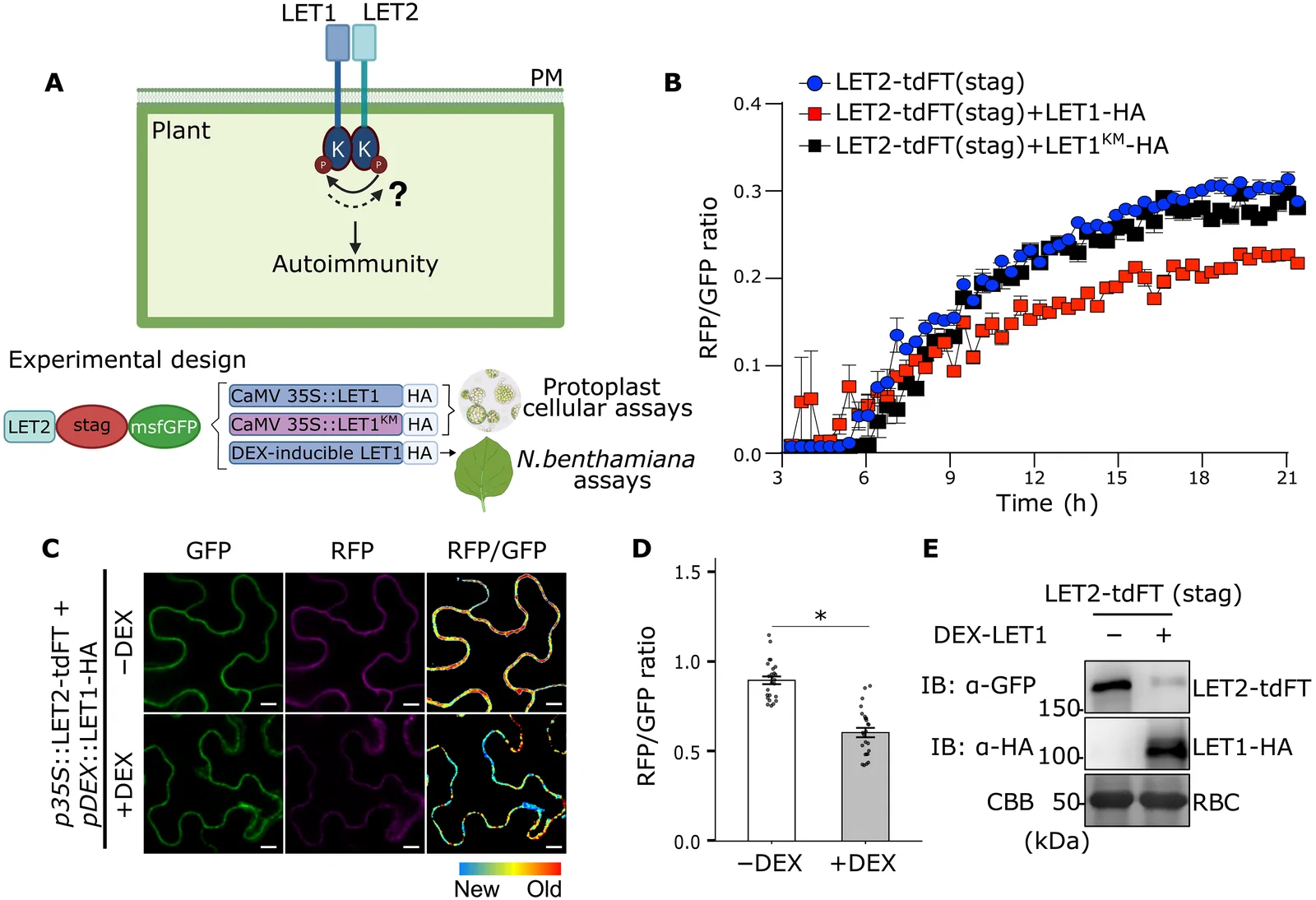
tdFT(stag) visualizes the protein stability of the PM receptor kinase LET2 in plant immunity signaling. (**A**) Upper panel: Scheme of LET1-LET2–modulated autoimmunity pathway. LET1 and LET2 associate with each other and trans-phosphorylate kinase domains. Lower panel: Experimental design for coexpression of the LET2-tdFT reporter and the LET1 effector proteins. (**B**) Coexpression of LET1 and LET2 in the *Arabidopsis* protoplast. LET1 promotes the turnover of LET2-tdFT(stag) in a kinase activity–dependent manner. LET2-tdFT(stag) turnover was accelerated under coexpression with LET1. Error bars indicate the means ± standard error (SE). *n* = 4. (**C** and **D**) Coexpression of LET2-tdFT and DEX-inducible LET1-HA in *N. benthamiana*. LET1 reduces lifetime and protein levels of LET2-tdFT in *N. benthamiana*. DEX (20 μM) was applied 48 hours after co-infiltration, and images were taken 6 hours posttreatment of DEX. Scale bars indicate 10 μm. Error bars indicate the means ± SE. *n* = 25. The asterisk shows significant difference between the samples (*P* < 0.05, Student’s *t* test). (**E**) Immunoblots showing that DEX-induced LET1-HA expression destabilizes LET2. Total extract was prepared from the samples in (C). RBC, ribulose bisphosphate carboxylase/oxidase large subunit.

The above analysis identified tdFT(stag) as the optimal FT for protoplasts, with a short initial lag time and a broad dynamic range suitable for the assay. We next applied this to investigate the turnover dynamics of the malectin-like receptor–like kinases LET1 and LET2. These PM-localized pattern-recognition receptors act upstream of intracellular nucleotide-binding leucine-rich repeat receptors (NLRs) to regulate immune signaling (*22*, *23*). LET1 and LET2 form a receptor complex, and LET2 trans-phosphorylates LET1 (Fig. 4A) (*24*), but the contribution of LET1 to LET2 protein stability remains unclear.

To test whether LET1 modulates LET2 turnover, we coexpressed LET2-tdFT(stag) with or without LET1-HA or the LET1 kinase-dead mutant (LET1^KM^-HA) in protoplasts and quantified RFP/GFP ratios as indicators of LET2 stability (Fig. 4, A and B). Coexpression with LET1, but not LET1^KM^, decreased the RFP/GFP ratio relative to LET2 alone, indicating reduced LET2-tdFT maturation in the presence of LET1 but not LET1^KM^ (Fig. 4B). These data suggest that LET1 promotes the turnover of LET2 in a kinase-dependent manner. Consistent trends were also observed in *Nicotiana benthamiana*, in which induced expression of LET1 by dexamethasone (DEX) led to a reduced RFP/GFP signal of LET2-tdFT(stag), coinciding with the decrease in overall protein levels detected by immunoblotting, confirming that LET1 promoted LET2 turnover (Fig. 4, C and D). Immunoblot analysis further showed that LET2-tdFT(stag) protein levels were reduced upon induced expression of LET1-HA, corroborating the RFP/GFP fluorescence ratio changes monitored by tdFT (Fig. 4E). Together, these results reveal that both the cell-autonomous protoplast expression system and *N. benthamiana*–based heterologous transient expression system are suitable to monitor the protein stability and longevity dynamics of *Arabidopsis* PM-resident receptor–like kinases regulated by interacting components in the complex.

### Evaluation of tdFT in stable transgenic plants with an inducible promoter

**Fig. 5.**
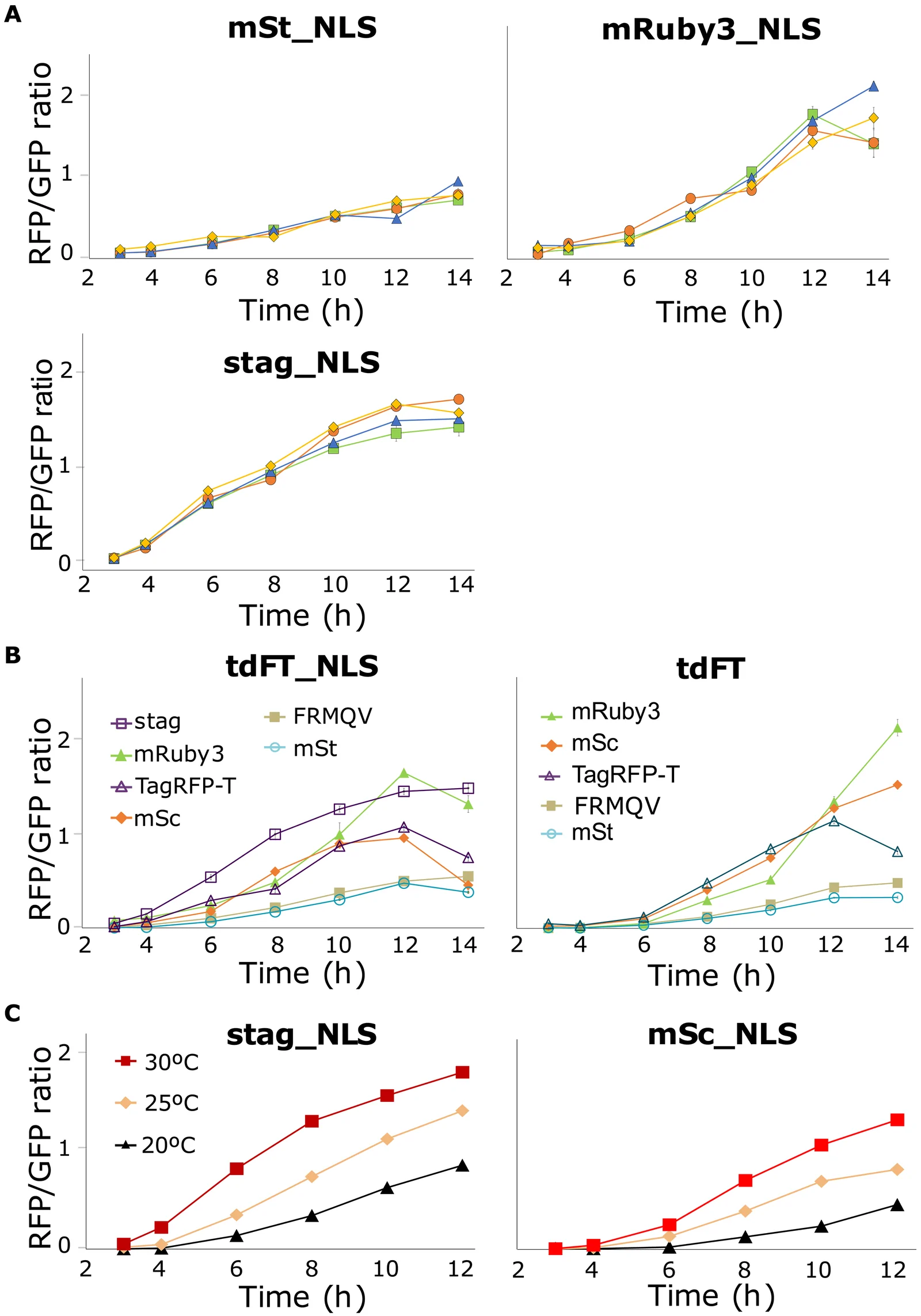
Maturation kinetics of inducible tdFT with various RFP variants. (**A**) Variation of RFP/GFP ratio among independent lines. Three to four lines were shown per construct. *n* = 15. (**B**) RFP/GFP ratio of cytosolic- and nuclear-targeted tdFT. (**C**) RFP/GFP ratio of tdFT-NLS (mSc and stagRFP) at different temperatures, 20°, 25°, and 30°C. *n* = 20. Error bars indicate the means ± SE.

Because many biological processes in plants and other organisms occur in tissue- and cell-specific manners, it is important to understand the feasibility and constraints of tdFT applications, as well as the observation-time windows of different tdFT configurations in intact plants. For this purpose, we prepared stable *Arabidopsis* transgenic plants with tdFT expression cassettes. In this case, tdFT expression is regulated by a β-estradiol–inducible promoter, allowing synchronized expression of tdFTs upon application of β-estradiol. *Arabidopsis* homozygous T3 lines with cytoplasmic- or nuclear-localized tdFT reporters were used for the analyses. First, we evaluated line-to-line variation of selected tdFTs [mSt, mRuby3, and stag with nuclear localization signal (NLS)]. Analysis of four independent transformants for each revealed that the kinetics of the ratiometric value showed little variation among independent transgenic events (Fig. 5A). This indicated that the intrinsic brightness and maturation time of each tdFT configuration could be visualized as a unique time course of RFP/GFP ratio development, even though expression levels of tdFT in each line/treatment could vary.

Next, the time course of RFP/GFP ratio for six tdFT configurations was analyzed. The analysis used nuclear-targeted tdFT-NLS to facilitate microscopic image data processing, and a subset of the data was confirmed using cytoplasmic tdFT as well. The ratiometric values typically reached a peak at 12 to 14 hours after β-estradiol induction for all RFPs, with the exception of the cytoplasmic tdFT(mRuby3). Data collected after 12 hours were not used in this analysis because of the unstable GFP and RFP signals that start to become problematic at 14 hours after induction. The shorter lag time (state 1, as short as 4 hours) observed in the stable transgenic plants than in the protoplasts was consistent with the presence of the transgenes already in the plant genome. We grouped tdFT into three categories on the basis of their line graph patterns (Fig. 5B). stag was categorized into class 1 because of its largest dynamic change in ratiometric value among all tested RFPs. Class 2 included mRuby3, mSc, and TagRFP-T, which showed an intermediate dynamic range. Class 3, comprising FRMQV and mSt, exhibited the smallest ratiometric dynamic range. The result mostly agreed with the protoplast-based classification, except mSt and TagRFP-T showing slower signal development in plants than in protoplasts. To determine the relative efficacy of different generations of tdFTs, we made a side-by-side comparison of estradiol-induced tdFT(mCh), tdFT(mSt), and tdFT(mSc) (fig. S2A). Under the same growth and detection condition, we found that the red signal from tdFT(mCh) became detectable earlier than mSt and mSc, and the RFP/GFP ratio became plateau 8 to 10 hours after the induction. The tdFT(mSt) ratio became plateau at 12 to 14 hours, and the tdFT(mSc) ratio continued to increase until 14 hours after induction. Again, data acquisition had to be stopped at 14 hours because of the instability of fluorescence signals. We concluded that the time range that can be analyzed by tdFT(mCh) is similar but slightly shorter than that by tdFT(mSt). This observation was consistent with the previously published maturation time.

Given that the maturation kinetics of fluorescent proteins are temperature-dependent and plant growth experiments are often conducted between 20° and 30°C, we measured the maturation kinetics of class 1 tdFT(stag) and class 2 tdFT(mSc) at different temperatures. As shown in Fig. 5C, the slope of the time-course curve steadily increased with increased incubation temperature. Notably, a substantial increase in RFP/GFP ratio was detected in tdFT(stag) 4 hours after induction. This result reinforced the importance of conducting a tdFT assay at the consistent temperature to secure data reproducibility.

### tdFT timers reveal differential distribution of aged proteins during cellular development

The above results successfully visualized the time course of tdFT maturation in plant cells using overexpression and inducible expression systems. We next applied selected tdFTs to visualize protein ages in developmental processes, where genes are temporally regulated, and proteins are dynamically controlled to govern stage-specific events. Here, the expression of reporter genes with an endogenous promoter is essential, although the analysis could be challenging often because of the weak and nonsynchronized expression of native promoters. The development of stomatal lineage cells initiates from a single-cell status followed by sequential cell division and differentiation events, each defined by unique cell morphology and the expression of specific marker genes (*25*–*27*). The *Arabidopsis* BREAKING OF ASYMMETRY IN THE STOMATAL LINEAGE (BASL) scaffold protein establishes cell polarity during the asymmetric division of stomatal lineage cells (*28*). Its precise localization and timing of action determine the orientation of cell division and the distinct fates of the resulting daughter cells (*29*, *30*). During stomatal development, although BASL proteins first appear in nuclei and subsequently in a polarized PM site in precursor cells, the cell cortical accumulation of BASL could result from active transportation from the nucleus or being newly synthesized in the cytoplasm. Here, tdFT-BASL was used to determine how the dual localization of BASL is achieved in stomatal lineage cells. We prepared several native promoter–driven tdFT-BASL configurations and introduced them into the *basl-3* mutant. tdFTs with mSt, mSc, and TagRFP-T produced green and red signals and complemented the *basl-3* mutant phenotype; however, the ones with mOr2 and mRuby3 did not produce an RFP signal, even though GFP signals were present. tdFT(stag)-BASL produced abnormal localization patterns. Apparently, fusing some RFP to BASL may interfere with the proper BASL or RFP function.

**Fig. 6.**
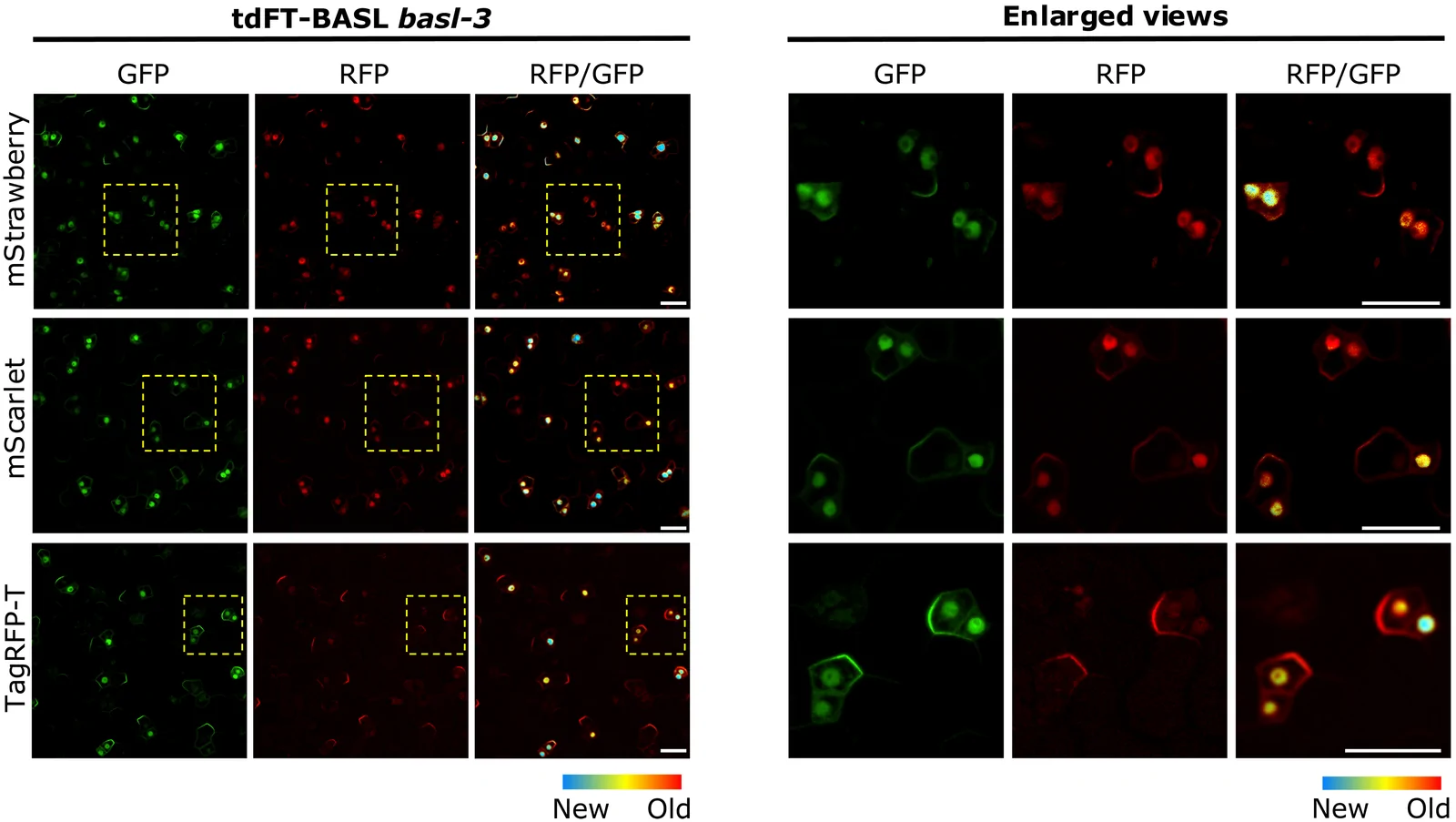
tdFT-based dual-color reporters reveal in vivo dynamics of BASL using different RFPs. Representative confocal laser-scanning microscopy images of *Arabidopsis basl-3* mutant plants transformed with *BASLpro*::tdFT(mSt)-BASL, *BASLpro*::tdFT(mSc)-BASL, and *BASLpro*::tdFT(TagRFP-T)-BASL. Images on the left panels show the adaxial surface of cotyledons from 2.5-day-old plants. Green: msfGFP; red: mSt, mSc, and TagRFP-T. RFP/GFP, ratio of absolute fluorescence intensity. Yellow boxes mark regions that are shown as enlarged views in the right panels. Scale bars indicate 20 μm.

Figure 6 shows different localization profiles of successful tdFT-BASL proteins, i.e., mSt, mSc, and TagRFP-T, in young cotyledons. In all three configurations, GFP signals were observed in nuclei of the protodermal cells (PrCs), meristemoid mother cells (MMC), meristemoid cells (MCs), and stomatal lineage ground cells (SLGCs). The crescent-shaped polarized sites of BASL are visible in MMC and SLGC at the PM, as described in previous observations (*28*). While the msfGFP signals were consistent and robust, a greater diversity of RFP signals was observed between different tdFT-BASL configurations. Relatively strong nuclear RFP signals were observed in tdFT(mSc)-BASL and, to a lesser extent, in tdFT(mSt); however, almost no nuclear RFP signals were observed in tdFT(TagRFP-T)-BASL. RFP signals in crescent sites were observed in all configurations. Because msfGFP, TagRFP-T, and BASL are produced as a single fusion protein, crescent-specific labeling of tdFT(TagRFP-T)-BASL indicates an older age of proteins, strongly supporting polarized protein translocation from the nuclei to the PM sites as opposed to the degradation of aged nuclear BASL or de novo production and PM targeting of young BASL. Protein production of tdFT-BASL proteins in each stomatal cell type was also evaluated by the fluorescence intensity per area (fig. S3), and there was a clear trend of BASL proteins highly accumulating to the dividing active MCs. tdFT(TagRFP-T) appeared to have a high capacity in distinguishing the SLGC population, in which more RFP was observed (fig. S3).

**Fig. 7.**
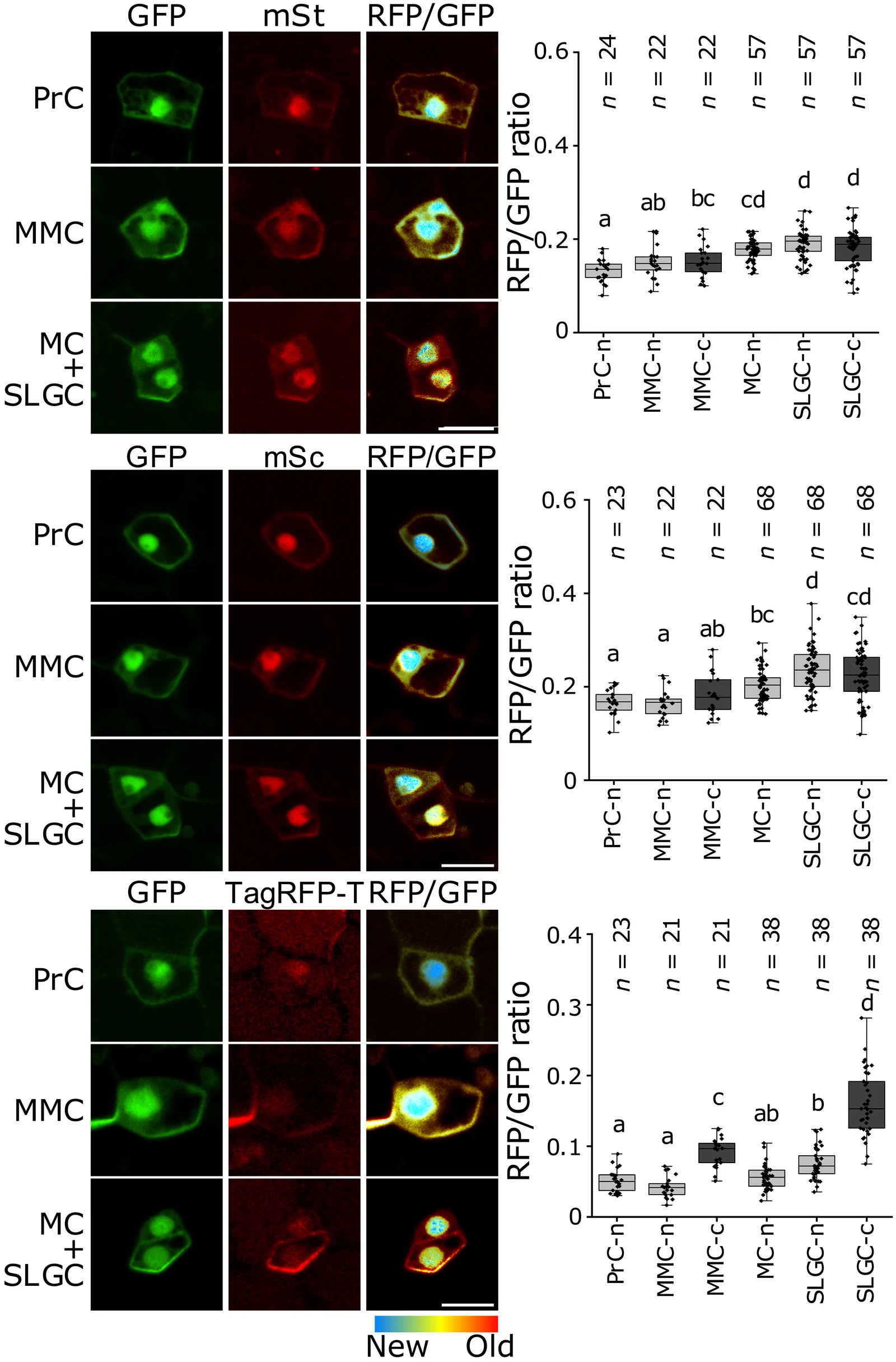
Intracellular distribution of tdFT-BASL reveals distinct trafficking events in different stomatal lineage cell types. Confocal images (left column) demonstrate the distribution patterns of GFP and RFP revealed by the tdFT marker lines, e.g., *BASLpro*::tdFT(mSt)-BASL (top), *BASLpro*::tdFT(mSc)-BASL (middle), and *BASLpro*::tdFT(TagRFP-T)-BASL (bottom). Scale bars indicate 10 μm. Box plots (right column) show quantification of RFP/GFP ratios. PrC-c, nucleus of the PrC; MMC-n, nucleus of the MMC; MMC-c, polarity crescent of the MMCs; MC-n, nucleus of the MC; SLGC-n, nucleus of the SLGC; SLGC-c, polarity crescent of the SLGC. Error bars indicate the means ± standard deviation (SD). Different letters indicate significant differences (*P* < 0.05) between genotypes [one-way analysis of variance (ANOVA) followed by Tukey’s post hoc test].

To establish the dynamics of BASL protein through early stomatal development, tdFT signals in subcellular domains were quantified in cells at different stages of stomatal development (Fig. 7). Overall, a gradual increase in tdFT aging (RFP/GFP ratio) was detected from PrC/MMC and MC to SLGC, particularly in tdFT(mSt)-BASL and tdFT(mSc)-BASL lines, indicating the likelihood of MC and SLGC inheriting the BASL protein expressed in MMC. Among the three tdFT configurations, tdFT(mSt) and tdFT(mSc)-BASL were less effective in differentiating BASL ages in the nuclei versus at the PM crescents; however, TagRFP-T clearly demonstrated an increased RFP/GFP ratio in the PM crescent over nuclei of the corresponding cells, indicating that the BASL protein likely translocates from nuclei to PM crescent sites. RFP signals from tdFT(TagRFP-T) were highly specific to PM crescent sites, indicating a substantially slow maturation of TagRFP-T in this analysis, contrasting with the profiles observed in protoplast and inducible expression systems (Figs. 2E and 5B).

### Limitation of tdFT and development of bpFT (KOR1 transport in the secretory system)

The tdFT-BASL trials described above showed successful visualization of protein ages in a process that occurs over a duration of a cell division cycle. Here, we tested whether the natively distributed protein age mosaic within the same generation of cells could be differentially visualized by tdFT-reporter proteins. For this test, we used *Arabidopsis* KORRIGAN protein that is synthesized in ER and accumulates in the trans*-*Golgi network (TGN), PM, and tonoplast (TP). Previously, we used inducible tdFT(mSt)-KOR1 reporters to track biogenesis and ab initio transport of KOR1 in the root meristem and visualized KOR1 delivery to PM and rerouting of mutated KOR1 from the PM to TP (*14*), establishing the basic route of KOR1 transport at the cell level. On the basis of this knowledge, we used native promoter–driven tdFT-KOR1 to visualize the steady-state distribution of protein ages in subcellular domains and gain insight into KOR1 homeostasis in planta.

**Fig. 8.**
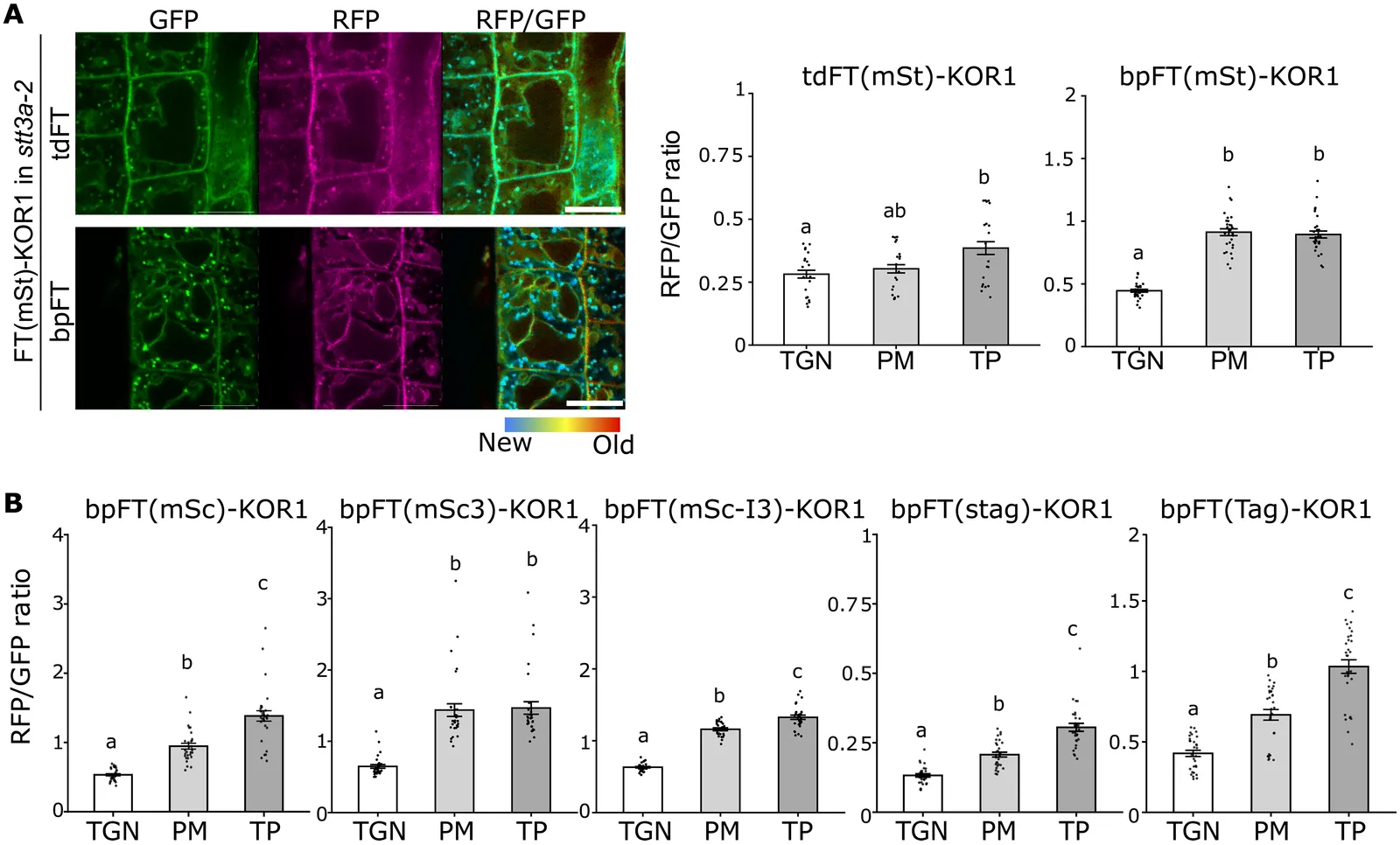
Ratiometric values of various *KOR1pro*::FT-KOR1 reporters at the TGN, PM, and TP. (**A**) Cellular RFP/GFP ratiometric values for tandem (tdFT-KOR1) and bipartite (bpFT-KOR1) constructs, each containing mSt, observed in *stt3a-2* root tip cells. *n* = 20. Scale bars indicate 10 μm. (**B**) Cellular RFP/GFP ratiometric values for bipartite (bpFT-KOR1) constructs using mSc, mSc3, mSc-I3, stag, and TagRFP-T (Tag), observed in *stt3a-2* root tip cells. Ratiometric values at the TGN, PM, and TP are presented as bar graphs. Error bars represent the SEM. *n* = 30. Different letters indicate significant differences (*P* < 0.05) between genotypes (one-way ANOVA followed by Tukey’s post hoc test).

On the basis of the previously successful tdFT(mSt)-KOR1 configuration, we prepared and introduced a KOR1 promoter–driven tdFT(mSt)-KOR1 into a *KOR1* mutant *rsw2-1*. The successful complementation of the *rsw2-1* root growth defect by this construct confirmed its functionality in planta (fig. S4). Subsequently, tdFT(mSt)-KOR1, and for comparison, tdFT(mCh)-KOR1, was introduced into the *stt3a-2* mutant, and the TGN-PM-TP distribution of tdFT-KOR1 age was quantified. The *stt3a-2* background promotes KOR1 trafficking to the TP resulting from KOR1 underglycosylation (Fig. 8A, upper micrograph, and fig. S2B) (*14*). Both tdFT(mSt)-KOR1 and tdFT(mCh)-KOR1 exhibited a higher RFP/GFP signal ratio in the TP compared to the TGN, indicating an older protein population in the TP. KOR1 in the PM showed an intermediate RFP/GFP ratio, and we were not able to separate the age of PM neither from TGN nor from TP with tdFT(mSt) (Fig. 8A), whereas the PM age aligned more with the TGN than TP in the case of tdFT(mCh). Overall, measurement windows of tdFT(mCh) and tdFT(mSt) were similar to each other, but tdFT(mCh) produced a steeper age gradient profile also in the native promoter system. During this analysis, we consistently observed slightly higher PM fluorescence intensity for tdFT(mSt)-KOR1 compared to GFP-tagged KOR1 (Fig. 8A, upper micrograph, and fig. S4A). Inconsistency in the intracellular protein distribution between GFP-KOR1 and tdFT-KOR1 could have been caused by a large size of tdFT inserted in the cytoplasmic domain, which may have affected protein traffic such as endocytosis, even though tdFT-KOR1 was functional enough to rescue the *rsw2-1* short root phenotype (fig. S4B).

To mitigate potential steric hindrance and further optimize the system, we developed an FT, bpFT. This system coexpresses individual msfGFP-KOR1 and mSt-KOR1 fusions under their own *KOR1* promoters in the same T-DNA (transferred DNA). This design compromises the single protein benefit of tdFT, but the gene dosage of msfGFP-KOR1 and mSt-KOR1 was kept 1:1, and reporter domains are as small as the monomeric fluorescent protein. The green channel signal of bpFT(mSt)-KOR1 successfully reproduced a localization pattern of single GFP-KOR1, demonstrating that the bpFT configuration did not interfere with the performance of individual XFP-KOR1 (Fig. 8A, lower micrograph, and fig. S4A). mSt-KOR1 showed very weak signals in the TGN but clear signals in the PM and TP, demonstrating that the maturation speed difference of msfGFP and mSt could be used to distinguish the age of KOR1 proteins in TGN and PM/TP compartments when expressed from the native promoter. However, we could not distinguish KOR1 ages in the PM and TP with bpFT(mSt)-KOR1.

To identify bpFT configurations suitable to distinguish KOR1 ages in multiple cellular compartments, a set of bpFTs was prepared. RFPs from class 1 and 2 classifications (stag, TagRFP-T, and mSc) as well as the bright RFPs mScarlet-3 (mSc3) and mScarlet-I3 (mSc-I3) were included (*31*). In stable transgenic plants, all bpFT configurations detected a protein age difference between the TGN and the PM/TP. However, only bpFT(mSc), bpFT(mSc-I3), and bpFT(TagRFP-T) were able to distinguish the TGN, PM, and TP (Fig. 8B). bpFT(mSc3) and bpFT(mSc-I3) produced consistent ratiometric signals, while bpFT(TagRFP-T) signals showed high sample-to-sample variabilities, perhaps due to low overall signal intensities of TagRFP-T. Unexpectedly, bpFT(stag) produced only weak overall RFP signals, with a higher level of signals in the vacuolar lumen. Given that different RFPs have distinct acid resistance, we did not include signals from the vacuolar lumen in this analysis, but this is an indication that KOR1 is ultimately incorporated into the vacuolar lumen (fig. S5).

### FT analysis in organ level protein aging

**Fig. 9.**
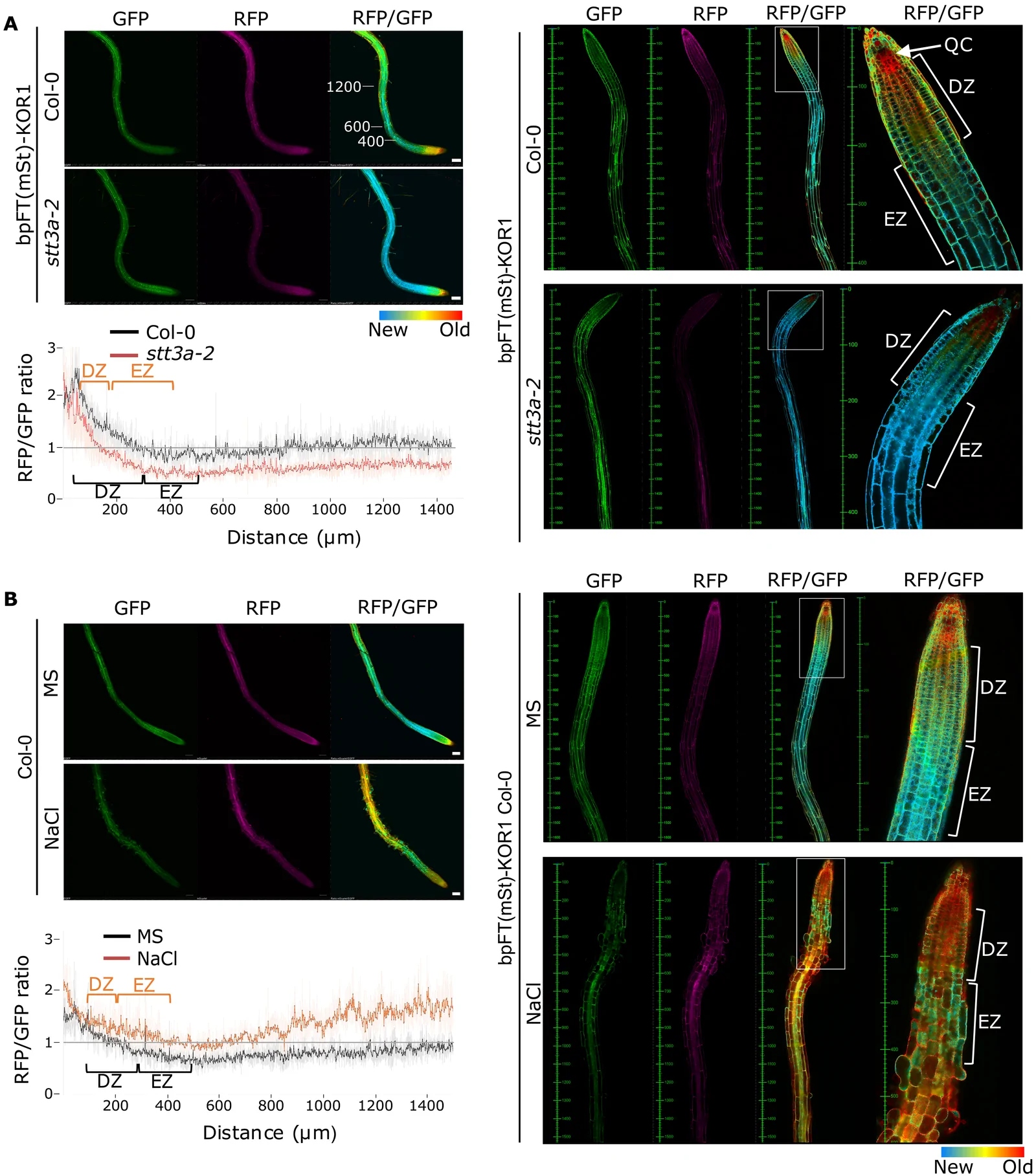
Spatiotemporal dynamics of KOR1 in the root. (**A**) Comparison of RFP/GFP ratio in *KOR1pro*::tdFT and bpFT(mSt)-KOR1 in Col-0 or *stt3a-2*. The numbers in the micrograph show the distance from the root tip. *n* = 5. Scale bars indicate 100 μm. (**B**) RFP/GFP ratio of *KOR1pro*::bpFT(mSt)-KOR1 in Col-0 with or without 140 mM NaCl for 24 hours. Three-day-old seedlings were used for these observations. The line graphs indicate the RFP/GFP ratio from the root tip toward the top of the roots along with a white arrow. Gray lines in the graphs indicate the level of RFP/GFP ratio = 1.0. DZ, division zone; EZ, elongation zone. *n* = 5. Error bars represent the SEM. The micrographs on the right show the detailed spatial pattern of the RFP/GFP ratio in root cells.

Enhanced TP targeting of KOR1 in the *stt3a* plants is a likely quality control mechanism to purge expired underglycosylated KOR1 proteins from the PM-TGN cycle for degradation in the vacuole. We investigated whether FT technology could differentiate protein ages at the organ level using bpFT-KOR1 expressed in the *Arabidopsis* root (Fig. 9A). In 3-day-old Col-0 background seedling, bpFT-KOR1 ratiometric profiles exhibited a high RFP/GFP ratio (aged) at the very end of the root, a decrease in KOR1 age toward the elongation zone (around 400 to 600 μm from the tip), and then gradual aging toward the base of roots. This suggested active production of young KOR1 and turnover of aged KOR1 in the cell division-cell elongation zones. Under high magnification, cells close to the stem cell niche contained aged KOR1, but cells in the division zone and elongation zone accumulated increasingly younger KOR1, forming a gradient (Fig. 9A, right panels). Notably, KOR1 in the root cap cells is younger than that in the inner cells, consistent with the high turnover of the root cap cells. (Note that red signals inside of vacuoles are autofluorescence.) In *stt3a-2*, the shape of RFP/GFP profiles was similar to Col-0; however, RFP/GFP ratiometric values were substantially lower in the entire profile, indicating that KOR1 in *stt3a-2* is overall younger than in Col-0. This is consistent with the model that underglycosylated KOR1 is unstable and more quickly turned over by TP targeting (Fig. 8B). When bpFT-KOR1 plants were exposed to the salt stress, there was an increase in RFP/GFP ratio, i.e., promoting aging of KOR1 in the cells (Fig. 9B). The salt response produced a steeper age increase in the tissue distal from the meristem, suggesting a decrease in KOR1 turnover under salt stress. Overall, bpFT could be a viable alternative when tdFT was not suitable. Together, FT can visualize the spatiotemporal “age” information of membrane proteins at both cellular and organ levels.

## DISCUSSION

Although tdFT systems have been developed and optimized in mammalian and yeast models, the implementation in plants has remained limited. In this study, we explored the potential of tdFT technology in monitoring plant proteins. One of the main challenges lies in identifying suitable fluorescence proteins whose maturation kinetics align with the dynamic range of plant protein turnover. While GFP derivatives mature relatively rapidly, the selection of an appropriate RFP that complements GFP and reliably reflects temporal changes in protein dynamics is critical for establishing functional tdFT systems in plants. To address this, we systematically evaluated multiple tdFT configurations across diverse experimental contexts, including high-throughput expression assays in *Arabidopsis* protoplast and stable transgenic *Arabidopsis* lines in which tdFT/bpFT reporters are driven by inducible or native promoters. Moreover, we here demonstrated the utility of the optimized tdFT system by generating tdFT/bpFT fusions with LET2, BASL, and KOR1, proteins that function in plant immunity, development, and abiotic stress adaptation. These applications underscore the versatility of tdFT reporters for monitoring protein dynamics in living plant cells and establish a framework that can be readily extended to a wide range of biological questions across plant sciences.

We tested first multiple combinations of RFPs with distinct maturation times and brightness, including FRMQV, mOr2, mRuby3, mSc, mSt, stag, and TagRFP-T, with GFP in *Arabidopsis* protoplasts, and quantified the RFP/GFP ratios. The cell-autonomous protoplasts are amenable to DNA transfections with nearly 100% cotransfection rates for different DNA species (*32*). Thus, characteristics of multiple POIs can be examined in a large population of living cells readily isolated from various *Arabidopsis* mutants and transgenic lines. Using transfected protoplasts coupled with the high-throughput plate reader system enabling quantification of GFP and RFP signals simultaneously in millions of cells, we were able to screen a series of tdFT configurations and select those suited for a wide range of research scenarios. Our investigation revealed that tdFTs with stag and mSc derivatives exhibited a relatively high dynamic range across the platform because of strong RFP signal intensities.

We next evaluate them in stable transgenic lines. In the native promoter system, mSc derivatives produced consistently high time resolution, and TagRFP-T showed distinct late maturation signals, albeit producing weak overall signals. In general, these RFP modules are reported to have high intrinsic brightness (≥60) (*31*, *33*, *34*). We concluded that RFPs with intrinsic brightness higher than mSt are potentially suitable for preparing tdFT. The performance of stag with native promoter–driven timers was not always consistent with transient and inducible promoter systems, making stag less suitable as a universal system. The tested tdFTs have been made available through Arabidopsis Biological Resource Center (ABRC) for interested parties to test in other systems. During our analysis, we unexpectedly found that reported parameters for maturation (*t*_0.5_) were rather unreliable when tdFTs were expressed in plants, except consistently very low signals from very slow RFPs, namely mOr2 (*35*) and FRMQV (*36*). In our tests, RFPs whose *t*_0.5_ ranges from 50 to 174 min produced comparable maturation kinetics (Figs. 2 and 5). This inconsistency could originate from the inconsistent experimental systems used to determine reported parameters but could also be caused by different impacts of lower temperature on the maturation of each RFP or by the unique environment in plant cells. We also suspect that the low intrinsic brightness of mSt and TagRFP-T (26.1 and 33.2, respectively) made apparent maturation slower because of the detection limit of RFP signals in our evaluation. When tested with native promoters, TagRFP-T produced a unique late-maturing profile specifically labeling “super-aged” compartments. Detection of super-aged compartments could be explored further using other class 3 RFPs like FRMQV.

Leveraging this methodological framework, we have sought to investigate the regulation of plant PM–localized receptor-like kinases, which sense endogenous and environmental cues to coordinate development and immunity. In many cases, receptor-like kinases and activated downstream signaling regulate intracellular NLRs, which coordinately maintain immune homeostasis (*37*). A well-established example is that the activated MEKK1-MKK1/2-MPK4 mitogen-activated protein kinase cascade suppresses NLR SUMM2 (SUPPRESSOR OF *mkk1 mkk2 2*)–mediated autoimmunity. Our earlier genetic and biochemical studies have identified several components, including LET1 and LET2, in regulating SUMM2-mediated autoimmunity (*23*, *37*, *38*), while in vivo cell biology studies about protein maturation, stability, subcellular localization, and interaction dynamics remain scarce. Using the selected tdFT configurations for real-time monitoring, we show that LET2 maturation is reduced in the presence of LET1, but not the catalytically inactive LET1^KM^, in both *Arabidopsis* protoplasts and *N. benthamiana* expression systems, consistent with the immunoblot results (Fig. 4, B to E). Given our previous finding that LET1 interacts with LET2 (*23*) and current observation that LET1 promotes LET2 turnover, we envisage that the reciprocal regulation between LET1 and LET2 contributes to immune homeostasis. Future work will be required to determine whether ubiquitination or additional posttranslational modifications mediate the LET1-LET2 regulatory module. In contrast to traditional end-point immunoblot assays, the tdFT system enables real-time visualization of protein maturation and turnover in vivo. Furthermore, combining tdFT reporters with protoplasts isolated from diverse genetic backgrounds provides a powerful strategy to dissect the contribution of individual signaling components to immune regulation. Last, by selecting tdFT configurations optimized for specific protein properties, such as subcellular localization or turnover rates, as demonstrated in our resource (Figs. 2E and 3 and fig. S1), this framework offers a flexible and scalable approach for monitoring immune complex dynamics in living plant cells.

In planta expression of reporter genes using a native promoter is required for protein visualization in the developmental process as well as in cellular processes that are sensitive to protein expression levels. Different timing of gene expression in diverse cell types in plants can substantially influence the choice of tdFTs. Cells in growing tissues are constantly replaced by a new population of cells and likely have a narrow observation window for protein ages than differentiated cells. Also challenging was the notion that the expression of a native promoter in a cell may not be as synchronized as the transient expression systems. Despite these challenges, we were able to visualize specific aging patterns in both the developmental process (stomata) and membrane protein transport in the root meristem, representing widely studied biological processes using native promoter–based systems. tdFT-BASL aging in the nuclei along stomatal differentiation, observed in all three timers examined (Fig. 7), suggested that a large portion of old molecules must be inherited from the precursor cells, despite the fact that new proteins are presumably synthesized after each round of mitosis. We also observed a differentially aged BASL protein in the MC and SLGC nuclei, only the latter of which contain the crescent portion and, accordingly, more aged nuclear pool. This likely resulted from either cell size–based molecular partitioning or active nucleocytoplasmic trafficking in the larger SLGC. At the subcellular level, although there was a similar trend, the TagRFP-T timer successfully demonstrated that BASL is substantially older at the polarity site than in the nucleus in both MMC and SLGC (Fig. 7), supporting the idea that BASL polarization involves an active nuclear export, a process requiring mitogen-activated protein kinase–mediated phosphorylation (*29*, *39*).

The analysis of tdFT-KOR1 in the root meristem cells provided an opportunity to assess the interaction of several parameters in the common platform. tdFT(mSt) was able to differentiate KOR1 ages in the TGN, PM, and TP when expressed by inducible expression but not when it was expressed by the native KOR1 promoter. This could be caused in part by fast maturation of mSt and slow PM-to-TP translocation of tdFT-KOR1 during biogenesis, an obstacle that required strongly synchronized induction and production of sufficient TP signals before mSt maturation to overcome it. With the native KOR1 promoter, it was necessary to use bpFT and use bright and slow RFPs like mSc, mSc-I3, and TagRFP-T to differentiate KOR1 ages in different subcellular compartments in the root meristem. At the organ levels, tdFT(mSt) and bpFT(mSt) produced similar age profiles and visualized more aging of KOR1 in Col-0 than in *stt3a-2*. This indicated the higher stability of KOR1 in Col-0 and, by contrast, more rapid turnover of aged KOR1 in *stt3a-2*.

We demonstrated that tdFT and bpFT could visualize protein ages in a wide range of plant applications. The FT reporter can be implemented in a research setting where fluorescent proteins are currently used without substantially altering the existing protocols. Together with previous studies, the analysis time scale of FT applications can span from 4 to 6 hours to several days. In our analyses using transient expression and native expression in actively growing/developing tissues, ages of proteins produced within a single cell division cycle are expected to be up to 35 hours, considering that the cell cycle duration time (*40*) and FTs with relatively fast-maturing RFPs performed well. On the other hand, proteins inherited from the progenitor cells or proteins produced in differentiated tissues could be older, and TagRFP-T with slow maturing behavior successfully labeled highly matured BASL protein populations. TagRFP-T and class 3 RFPs, which were not favored in this study, are potentially useful for expanding timer ranges for long-life-cycle proteins. mOr2 has been successfully used in mammalian cell–optimized FT (*7*).

Several limitations of FT analyses became apparent in this study. Because of its dimeric nature, tdFT is a relatively large molecule, requiring careful consideration of the tagging site in the protein. In this study, tdFT placed in the cytoplasmic domain of KOR1 altered KOR1 dynamism at the PM, even though tdFT-KOR1 was able to complement the *rsw2-1* mutant. Also, we were not able to target tdFT into the luminal side of post-ER compartment because tdFT was retained in ER even with a vacuolar targeting signal (Fig. 3). Because of this limitation, we did not test FT functionality in the Golgi apparatus, TGN, endosome, apoplast, and vacuole. A workaround for these problems was to use bpFT. In using bpFT, it was important to express both GFP and RFP reporter proteins from the same T-DNA cassette so that the gene dosages are kept equal. Furthermore, when the inside of the lumen of post-ER compartments becomes progressively acidic (*41*) and substantially decreases fluorescence intensity (*42*), the use of acid-resistant FT configuration may become necessary. Because of the availability of a constantly developing, wide variety of GFP and RFP modules, it is likely that proper FT configurations are available for many plant applications.

Preceding studies successfully applied tdFT technology in soluble protein turnover by a ubiquitin-proteasomal pathway as well as transport of de novo–synthesized membrane proteins (*13*, *14*). This study expanded the application success to the steady-state turnover of membrane proteins, steady-state transport of membrane proteins, and developmentally regulated polar protein transport, demonstrating a wide application potential of the tdFT and bpFT technologies. Potential future applications could include, but are not limited to, visualization of dynamism in protein translocation from the PM to TPs (*43*), hormone-induced receptor turnover (*44*), longevity of factors in the developmental signal cascade in stomatal lineage cells (*45*), and longevity of protein complexes (combined with bimolecular fluorescence complementation technologies). Also interesting is monitoring the combined transcriptional and posttranslational regulation of auxin [indole-3-acetic acid (IAA) proteins] and jasmonate [jasmonate ZIM-domain (JAZ) proteins] signaling, where induction of gene expression coincides with destabilization of protein products (*46*, *47*). Even though hormone destabilization of IAA and JAZ proteins has been shown using 35*S* promoter–driven tdFT-IAA (*13*) and GFP-JAI3(JAZ) (*47*) previously; however, their native promoters, which are induced by auxin and jasmonate, respectively, can produce substantially different protein dynamics of endogenous proteins in the hormone-treated plants. Beyond individual protein dynamics, the FT technology may be applicable to labeling cell ages in the population, distinguishing slow and fast cycling cells in the meristem, the rate of vesicle transport, and local versus long-distance protein signals. The technology could be further improved by inserting the FT module into genomic loci using CRISPR knockin (*48*). Furthermore, the high-throughput protoplast transient system could be developed to screen a chemical library or panel of expression clones to identify chemical or protein effectors affecting POI turnover and other spatiotemporal dynamics.

## MATERIALS AND METHODS

### Plant materials and growth condition

*Arabidopsis thaliana* Col-0 wild-type and *rdr6-11, stt3a-2* mutant lines and *N. benthamiana* were used for this study. For protoplast preparation from *A. thaliana* Col-0 and agroinfiltration, plants were grown in soil (Sungro horticulture sunshine mix no. 5) in a growth room controlled at 23°C, 45% relative humidity, and 100 μE m^−2^ s^−1^ light with a 12-hour light/12-hour dark photoperiod. For flower transformation of *A. thaliana*, wild-type and mutant plants were grown in soil (Jolly Gardener C/20 potting mix) at 23°C in 16-hour light/8-hour dark condition. For in vitro culture, sterilized seeds were germinated on media containing 1/4× strength Murashige and Skoog salts, 0.5% sucrose, and 0.7 or 1.5% agar and were grown in an incubator set at 25°C with a 16-hour light/8-hour dark regime using 50% output of PHILLIPS F17T8TL741.

### Construction of tdFT expression cassettes

The detailed cloning procedure and primer sequences are described in the Supplementary Text and data S1. tdFT plasmids were constructed using the pEnEOimCherryF3SGT vector (GenBank accession number KF537341.1). N-tdFT cassettes were prepared in an msfGFP-RFP configuration, whereas C-tdFT was prepared in an RFP-msfGFP configuration. msfGFP and RFP plasmids were obtained from Addgene (nos. 29725, 58170, 105802, 54568, and 54586), and FRMQV and mSc were provided by R. Jimenez. The stag coding sequence was prepared with the QuikChange protocol using TagRFP-T as a template. The N-tdFT or C-tdFT sequence was inserted between Nco I/Sca I sites or Nco I/Not I sites in pEnEOimCherryF3SGT, respectively. The sequences of the resulting tdFT plasmids were deposited to the GenBank database (accession nos. OP013018-OP013025 and OR569798-OR569804), and plasmids were deposited to ABRC (CD3-2930 to CD3-2937 and CD3-2942 to CD3-2948). For protoplast transformation, C-tdFT coding sequences were amplified by polymerase chain reaction (PCR) and cloned into the pHBT vector (GenBank accession number EF090408) between Bam HI/Pst I sites under the control of a cauliflower mosaic virus (CaMV) 35*S* promoter. To introduce tdFT into stable transgenic plants, N-tdFT was amplified by PCR and inserted into the pER8ex vector (*14*) between Xho I/Spe I sites. For nuclear-targeted expression in transgenic plants, the 12-amino-acid sequence MKASRNKRLKPD–containing NLS of *Arabidopsis* CPL1 (At4g21670) was added to tdFT (TagRFP-T and stag) before the stop codon (short NLS). For tdFTs with other RFPs, a long NLS (nucleotides 2482 to 2904 of CPL1) was added because the short NLS was not effective.

### Targeting tdFT expression in intracellular organelles/domains

Expression constructs for subcellular organellar-targeted tdFTs were derived from pHBT-C-tdFT(stag). For plastid targeting, the transit peptide from rbcS (At1g67090, nucleotides 1 to 231) was inserted in the Bam HI site of pHBTC-tdFT(stag). For ER localization, the signal peptide from basic chitinase (At3g12500, nucleotides 1 to 60) was inserted at the Bam HI site and HDEL sequence before the stop codon. Similarly, for vacuolar targeting, the signal peptide and AFVY sequence were inserted at the Bam HI site and before the stop codon, respectively. For peroxisome targeting, the SKL sequence was inserted before the stop codon. For targeting tdFT to mitochondria, three sequences were tested. We tested the targeting peptides of the β subunit of *Arabidopsis* mitochondrial adenosine 5′-triphosphate synthase (At5g08680, nucleotides 1 to 273) and COX4 protein of *S. cerevisiae*, which was inserted in the Bam HI site of pHBTC-tdFT(stag). As an alternative approach, the full-length sequence of *Arabidopsis* IDH1 encoding mitochondrial isocitrate dehydrogenase was inserted at the Bam HI site.

For evaluating PM-localized protein behaviors in protoplasts, the *Arabidopsis LET2/MDS1* (*AT5G38990*) coding region was amplified by PCR using a primer pair M7/M8 and inserted into the Bam HI site of pHBTC-tdFT(stag) by Clonexpress (Vazyme Biotech). Similarly, the *LET1* (*AT2G23200*) coding region was amplified using a primer pair M5/M6 and inserted into the Bam HI site of pHBTC-tdFT(stag) and pHBT-HA. LET1^KM^ (K714E) was generated by site-directed mutagenesis by the QuikChange protocol (*49*) using primers M12/M13. For the *N. benthamiana* expression assays, the C-tdFT(stag-msfGFP) fragment amplified using a primer pair M1/M4 was inserted between Bam HI/Pst I sites of the pCAMBIA2300 vector to generate pCAMBIA2300-p35S::C-tdFT(stag-msfGFP) by Clonexpress (Vazyme Biotech). For generating pCAMBIA2300-p35S::LET2-C-tdFT, the LET2 coding region fragment amplified using a primer pair M7/M8 was inserted into the Bam HI site of the pCAMBIA2300-p35S::C-tdFT vector. For DEX-inducible LET1, LET1 was amplified by PCR from Col-0 cDNA using a primer pair M10/M11 and inserted between Xho I/Stu I sites of pTA7002-HA modified from pTA7002 (*50*) under the control of a CaMV 35*S*:GVG DEX-inducible promoter (pTA7002-pDEX-inducible-LET1-HA hereafter).

### Protoplast transient expression

The protoplast transient expression with pHBTtdFT constructs was performed as described previously (*32*) with modification. Briefly, 20 μl of plasmid DNA (2 μg/μl) was transfected with 200 μl of protoplast (2 × 10^5^ cells/ml) for organelle localization, and 25 μl of plasmid DNA (2 μg/μl) was transfected to 500 μl of protoplast for transfection efficiency quantification, plate reader analysis, and ratiometric analysis. For the microscope observation, a WI solution is modified as one without MES [0.5 M mannitol, 20 mM KCl, and 1 mM CaCl_2_ (pH 5.7)].

### Transient expression assay in *N. benthamiana*

For transient expression in *N. benthamiana*, a single colony of *Agrobacterium tumefaciens* GV3101 carrying either pCAMBIA2300-p35S::LET2-tdFTstag or pTA7002-pDEX-inducible::LET1-HA was cultured overnight at 28°C in 2 ml of LB liquid medium containing kanamycin (50 μg ml^−1^) and gentamicin (25 μg ml^−1^). Cells were harvested by centrifugation at 1200*g* for 10 min and resuspended in infiltration buffer containing 10 mM MgCl_2_, 10 mM MES buffer (pH 5.7), and 200 μM acetosyringone to OD_600_ (optical density at 600 nm) = 1.0. The suspensions were infiltrated into the leaves of 4-week-old *N. benthamiana.* DEX (50 μM) was applied by spraying or infiltrating to leaves 36 hours postinfiltration of agrobacterial cells, and leaf tissue was collected 5 hours later for protein isolation and tdFT fluorescence imaging. Images from a total of 25 cells were analyzed (five cells per leaf from five separate plants).

### Plate reader analysis

The fluorescence intensity of protoplasts expressing stagRFP-msfGFP constructs was quantified using a Cytation 7 Cell Imaging Multi-Mode Reader (BioTek Instruments, US) in the kinetic mode. Protoplasts were incubated at 25°C in the dark for 6 hours after transfection and then transferred into a 12-well black, clear-bottom plate for fluorescence measurement. Fluorescence readings were recorded every hour from 6 to 14 hours posttransfection to monitor the time course.

For an even suspension of protoplast, plates were gently shaken horizontally for 1 min at 567 cycles per minute (cpm) with a 3-mm amplitude. All readings were performed using the top optics configuration with a xenon flash lamp as the excitation source and extended dynamic-range gain settings. Fluorescence was read at a 7-mm height using a 100-ms delay and 10 repeated measurements per data point to minimize the background reflection and optimize signal detection. For GFP fluorescence, excitation and emission wavelengths were set to 485 ± 20 and 528 ± 20 nm, respectively, and each well was scanned using a 5 × 5 matrix area-scan mode to capture spatial fluorescence distribution. An additional area scan at 574 ± 20–nm excitation/528 ± 20–nm emission was included as a default Gen5 acquisition setting with the changed excitation wavelength only. This scan is for quality control to evaluate signal distribution across the entire well and to identify potential technical artifacts such as cell aggregation, uneven signal distribution, or signal distortion caused by debris or air bubbling adhering to the plate bottom under identical gain and exposure conditions. End-point fluorescence was then measured for both fluorophores using dual filter sets: 483 ± 20–nm excitation/528 ± 20–nm emission for GFP and 574 ± 20–nm excitation/620 ± 20–nm emission for RFP. Fluorescence data were collected up to 21 hours (the longest possible before drying of samples) and processed using Gen5 software (BioTek). For each time point and well, an average of 10 repeated readings was calculated to obtain a stable signal. Data were obtained from three or four biological replicates. The results were analyzed and presented as relative fluorescence units. The experiment was repeated at least twice.

### Confocal microscopy

Fluorescence imaging was performed on a Nikon AX (estradiol-inducible tdFT and td/bpFT-KOR1) or Leica TCS SP8 (protoplasts, LET1/LET2) confocal laser-scanning microscope and the Andor Dragonfly 610 system on Leica DMi8 (tdFT-BASL). GFP fluorescence was excited at 488 nm, and emitted light was detected between 490 and 530 nm. Various RFP proteins, FRMQV, mOr2, mRuby3, mSc, mSt, stag, and TagRFP-T, were excited at 566, 549, 558, 569, 574, 555, and 555 nm, respectively, with emission detected between 560 and 620 nm.

### Protein isolation from *Arabidopsis* protoplast and *N. benthamiana*

*Arabidopsis* protoplasts expressing the indicated constructs were resuspended with W5 buffer (at the same volume used for the original WI incubation), collected at 100*g* for 2 min, and lysed in 80 μl of 1× SDS loading buffer. For *N. benthamiana*, two leaf discs (0.5-mm diameter) from the infiltrated area were homogenized in 80 μl of 1× SDS loading buffer. All samples were boiled at 95°C for 5 min to elute protein.

### Immunoblotting

Denatured proteins (8 to 10 μl) were loaded onto 10% SDS–polyacrylamide gel electrophoresis gel and run at 80 V for 30 min through the stacking layer, followed by separation at 90 to 130 V until protein marker was clearly separated. Proteins in SDS–polyacrylamide gel electrophoresis gel were transferred onto the polyvinylidene difluoride membrane and blocked with 5% skim milk in phosphate-buffered saline with Tween 20 (PBST) for 40 to 60 min at room temperature. The membrane was incubated overnight at 4°C with rat anti–HA-Peroxidase (Roche, cat. no. 12013819001; RRID: AB_390917) or mouse anti-GFP antibody (Roche, cat. no. 11814460001; RRID: AB_390913) diluted to 1:2000 in PBST, and after three PBST washes, the GFP-blot was probed for 1 hour at room temperature with the anti-mouse IgG HRP-linked antibody (Cell Signaling, cat. no. 7076; RRID: AB_330924) diluted to 1:10,000 in PBST, followed by three PBST washes. Blots were then developed by enhanced chemiluminescence. The experiment was repeated at least twice.

### Preparation of transgenic plants and tdFT data acquisition and analysis

pER8extdFT plasmids were introduced into *A. tumefaciens* ABI and used for transforming the *A. thaliana* Col-0 *rdr6-11* host (*14*). Transgenic plants were selected by hygromycin resistance, and data were obtained from T2 and T3 lines, except for T1 plants for tdFT(mCh). For induction of tdFT expression, transgenic plants were grown on vertical plates with solid medium containing 1× MS salts, 3% (w/v) Suc, and 1.5% (w/v) agar for 5 days. Plants were transferred to fresh agar plates and covered with 1 ml of induction solution [0.4 μM β-estradiol in 1× MS salts and 3% (w/v) Suc], which was infiltrated by brief application of the vacuum. Plants were incubated for 2 hours for transgene induction and subsequently transferred to fresh medium without β-estradiol until observation to avoid excess and delayed expression. Fluorescence images were acquired using a C1si confocal microscope equipped with NIS Elements confocal microscope software (NIS Elements, Nikon) and processed by NIS Elements.

Data were collected until 14 hours postinduction, at which time the induced fluorescence signals had receded close to the background levels and robust RFP/GFP ratio measurements were no longer possible; thus, imaging was limited to 14 hours. Data were collected from 20 to 25 cells for each data point. The experiment was repeated at least twice.

### Preparation of tdFT-BASL transgenic plants and data analysis

tdFT-BASL expression clones were prepared on the basis of pMDC43-pBASL-GFP-BASL (*39*). First, the Kpn I/Bbv CI fragment was replaced with a PCR product amplified with a primer pair 1796/1800 encoding the genomic fragment of BASL, including the first exon, the first intron, and the second exon. Then, the N-tdFT(mSt) coding sequences amplified by a primer pair 1768/1799 were inserted into Kpn I/Bam HI sites. Then, the mSt sequence was replaced with mSc and TagRFP-T amplified using primer pairs 1912/1799 and 1913/1916. Resulting pMDCtdFT-BASL clones were introduced into GV3101 and transformed into *basl-3* mutant lines by flower transformation.

Three independent transgenic lines were obtained: *BASLpro::tdFT(mStrawberry)-BASL*, *BASLpro::tdFT(TagRFP-T)-BASL*, and *BASLpro::tdFT(mScarlet)-BASL*. Confocal images were collected as described above. Quantitative image analysis was conducted using Fiji ImageJ. Fluorescence intensities of GFP and RFP were measured from confocal images in different stomatal lineage cell types, including PrCs, MMCs, MCs, and SLGCs. For each cell, the total fluorescence intensity and the mean intensity normalized to unit area were calculated (fig. S3). In Fig. 7, the red-to-green fluorescence ratio was calculated on the basis of the relative intensities in the nucleus and crescent regions of different cell types to evaluate BASL polarity behavior. The data were collected from more than 10 independent plants for each genotype. The experiment was repeated at least twice with similar results.

### Preparation of bipartite FT-KOR1

Gateway entry clones pEnmsfGFP-KOR1 and pEnRFP-KOR1 were prepared by inserting the msfGFP and RFP coding sequence into the Bam HI site of pEnRGSHis-KOR1 (*14*). pEnmsfGFP-KOR1 was recombined with pMDC99 using LR clonase II to generate pMDC99-msfGFP-KOR1. A gateway cassette was then introduced into the Pme I site. Resulting pMDC99-msfGFP-KOR1-GW was used to introduce the RFP-KOR1 cassette to produce the pMDC99-msfGFP-KOR1-RFP-KOR1 (pMDC99GKRK) series. pMDC99GKRK plasmids were introduced into GV3101 for flower transformation of *A. thaliana* Col-0.

### Ratiometric image quantification and presentation using Fiji

The ratiometric images in Figs. 2D, 4C, 6, and 7 and fig. S1A were generated using Fiji software. GFP and RFP image files were first converted to 32-bit format. A ratiometric image was created using the Calculator Plus function with RFP as *i1* and GFP as *i2*, applying the division operation (K1 = 1 and K2 = 0). A custom lookup table was applied. Brightness and contrast were initially adjusted, followed by conversion of the ratiometric image from 32-bit to RGB (red, green, and blue) color and subsequently to hue, saturation, and brightness (HSB) stack. The brightness channel in the HSB stack image was removed and replaced with the GFP image. The modified image was then converted back to RGB format, and final brightness and contrast adjustments were performed to optimize visualization.
