# Supplementary material for: A suite of fluorescent timers for visualizing protein spatiotemporal dynamics in plants
Source: Sci Adv. 2026 Sep 16;12(38):eaef3538. doi: 10.1126/sciadv.aef3538 (PMC13580541; doi:10.1126/sciadv.aef3538)
Supplement: Supplementary file 1 [file sciadv.aef3538_sm.pdf]

Science Advances
AAAS

Supplementary Materials for

A suite of fluorescent timers for visualizing protein spatiotemporal dynamics in plants

Yukihiro Nagashima *et al.*

Corresponding author: Hisashi Koiwa, hisashi.koiwa@ag.tamu.edu

Sci. Adv. **12**, eaef3538 (2026)
DOI: 10.1126/sciadv.aef3538

The PDF file includes:

Supplementary Text
Figs. S1 to S5
Legend for data S1

Other Supplementary Material for this manuscript includes the following:

Data S1

Supplementary Text: Detail of cloning procedures

Basic tdFT constructs

A new set of N-tdFTs were prepared using previously prepared tdFT vector pEnEOimsfGFP-mStF3SGThsp (pEnEOitdFT(mSt)). mStrawberry coding sequence was excised by digestion with AscI and ScaI and replaced with PCR fragments encoding different RFPs. RFP coding sequences were amplified using primer pairs, mOrange2 (1912/1939), mScarlet (1912/1939), mRuby3 (1913/1938), FusionRedMQV (1915/1937), tagRFP-T (1913/1937). Both the pEnEOimsfGFP-mStF3SGThsp vector and the PCR products were digested by AscI/ScaI and ligated to produce new N-tdFT expression plasmid series. tdFT(stag) was prepared by quickchange protocol using a arimer pair (2041/2042)

C-tdFT vectors were prepared by digesting pEnEOitdFT(mSt) with ScaI/NotI and inserted the 2nd copy of GFP sequence amplified with M14/M15 using Clonexpress (Vazyme Biotech) resulting in pEnEOimsfGFP-mSt-msfGFPF3SGThsp. Subsequently, a unique BamHI site was introduced by quickchange protocol using primers M16/M17. msfGFP-mSt fragment was then excised by NcoI/ScaI digestion and replaced with PCR products corresponding to different RFPs amplified using primer pairs M18/M19 (mStrawberry and mOrange2), M20/M19 (mScarlet), M21/M22 (mRuby3), M21/M23 (tagRFP-T and stag), and M24/M25 (FusionredMQV). Resulting tdFT CDS were amplified by PCR using primer pairs (M1/M2, M1/M3, M1/M4) and cloned into pHBT-HA digested with BamHI/PstI.

Inducible tdFT were prepared by ligating XhoI/SpeI-digested pER8ex and N-tdFT coding sequences. tdFT fragments were amplified by PCR using primer pairs: mOrange2 (1943/1939), mScarlet (1943/1939), mRuby3 (1943/1938), FusionRedMQV (1943/1937), tagRFP-T (1943/1937) and mCherry (1943/2619).

The Nuclear-localized tdFT(tagRFP-T) in pER8ex vector were prepared by amplifying tagRFP-T-NLS sequence by PCR using a primer pair (2022/2054) using pEnEOitdFT(tagRFP-T) as template. pER8extdFT(tagRFP-T) and PCR productes were digested with AscI/SpeI and ligated to produce pER8extdFT(tagRFP-T)NLS. pER8extdFT(tagRFP-T)NLS was then digested with AscI/BspHI and ligated with PCR products amplified using primer pairs 2022/2046 (mStrawberry, mScarlet, and mOrange2), 2022/2047 (mRuby3), 2022/2048 (stag), and 2022/2049 (FusionredMQV). pER8extdFT(stag)NLS and pER8extdFT(stag)NLS were used for the analysis. pER8extdFTNLS with other RFPs were further modificed by replacing the short NLS sequence with longer NLS. CPL1 NLS wsequence was amplified by PCR using a primer pair (2069/2070) and CPL1 cDNA as template. The NruI/SpeI digested pER8extdFTNLS were ligated with SpeI digested PCR product to produce pER8extdFTNLS for mOR, mSt, mSc and mRuby3.

Targeting tdFT expression in intracellular organelles/domains

Expression constructs for subcellular organellar targeted tdFTs were derived from pHBT-C-tdFT(stag). For plastid targeting, transit peptide from rbcS (At1g67090, 1-231 nucleotide) was amplified by PCR (primer pair 2220/2221) and inserted in the BamHI site of pHBTC-tdFT(stag). For ER localization, signal peptide from basic chitinase (At3g12500, nucleotide 1-60, primer pair 2218/2219) was inserted at the BamHI site and then HDEL sequence (annealed oligonucleotides 2212/2213) before the stop codon using Clonexpress (Vazyme Biotech). Similarly, vacuolar targeting, inverse PCR using pHBTsignalpeptide-tdFT signal peptide using primer pair N1/N2 and AFVY sequence (annealed oligonucleotides 2214/2215) were recombined using Clonexpress (Vazyme Biotech). For peroxisome targeting, SKL sequence (annealed oligonucleotides

2216/2217) was inserted before the stop codon using Clonexpress (Vazyme Biotech). For targeting tdFT to mitochondria, three sequences were tested. We tested targeting peptides of beta subunit of Arabidopsis mitochondrial ATP synthase (At5g08680, 1-273 nucleotide, primer pair 2222/2223) and COX4 protein of *Saccharomyces cerevisiae*). Products of the 1st thermal cycling (10 cycles) using oligonucleotides 2274/2275/2276 were used as the template for the 2nd PCR using primer pair 2277/2278, which was then inserted in the BamHI site of pHBTC-tdFT(stag) using Clonexpress (Vazyme Biotech). As an alternative approach, full-length sequence of Arabidopsis *IDH1* encoding mitochondrial isocitrate dehydrogenase was amplified by PCR using primer pair 2394/2395 was inserted at the NcoI site of pEnEOistag-msfGFP. Inserting *IDH1* in pHBT vector caused a cell toxicity in *E. coli* and was not used.

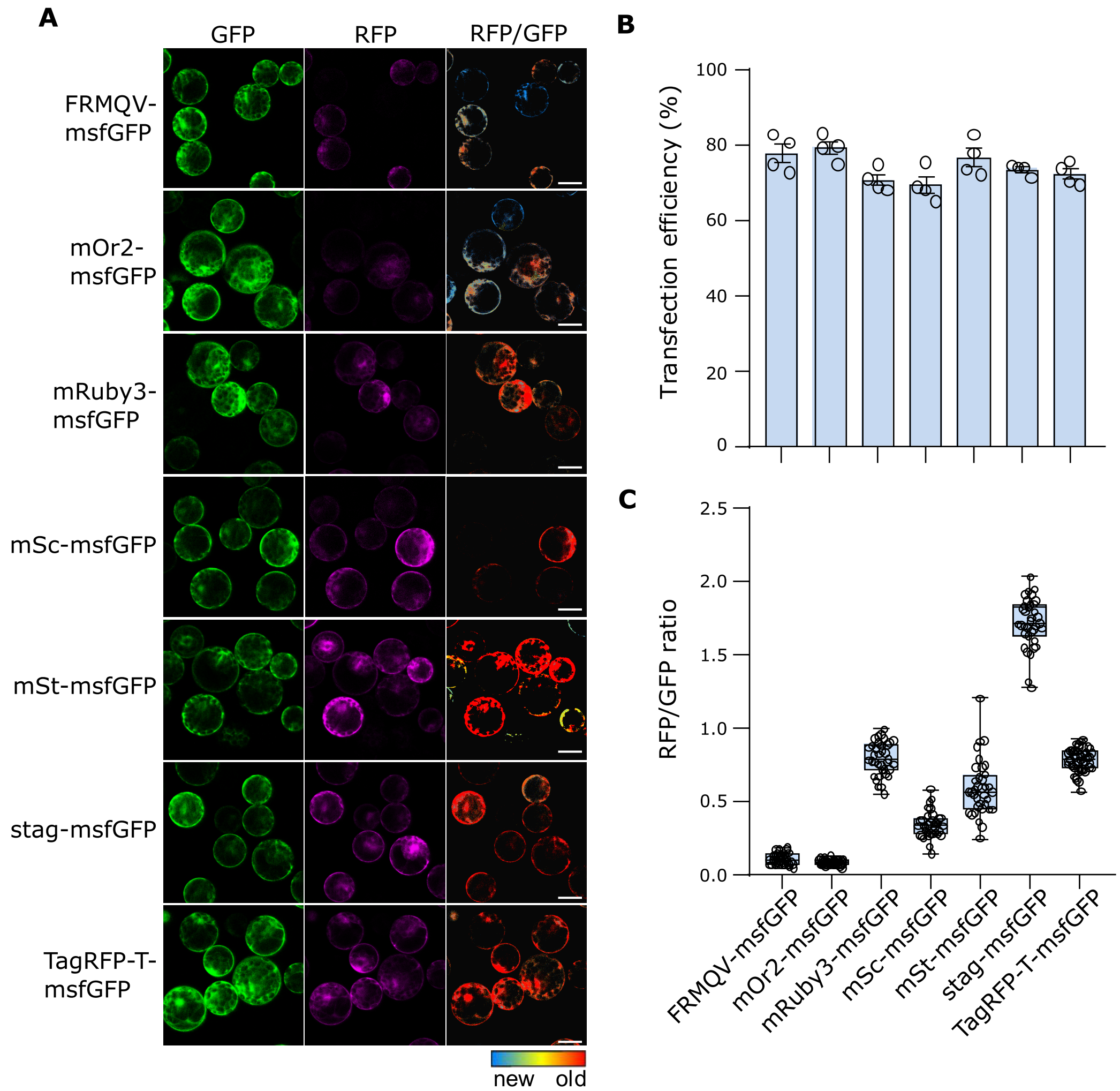

Fig. S1. Application of tandem fluorescent timers (tdFT) for dynamic analysis in Arabidopsis protoplasts.

(A) Confocal images of tdFT with different RFP variants in the Arabidopsis protoplast transient expression system. Scale bars are 25 µm. (B) tdFT with different RFP variants in the Arabidopsis protoplast transient expression system shows comparable transfection efficiency. *n* = 4. (C) Comparison of tdFT with different RFP variants in the Arabidopsis protoplast transient expression system. The optimal combination for the tdFT assay was identified as stagRFP and msfGFP. *n* = 36-54. Error bars represent the standard error of the mean (SEM).

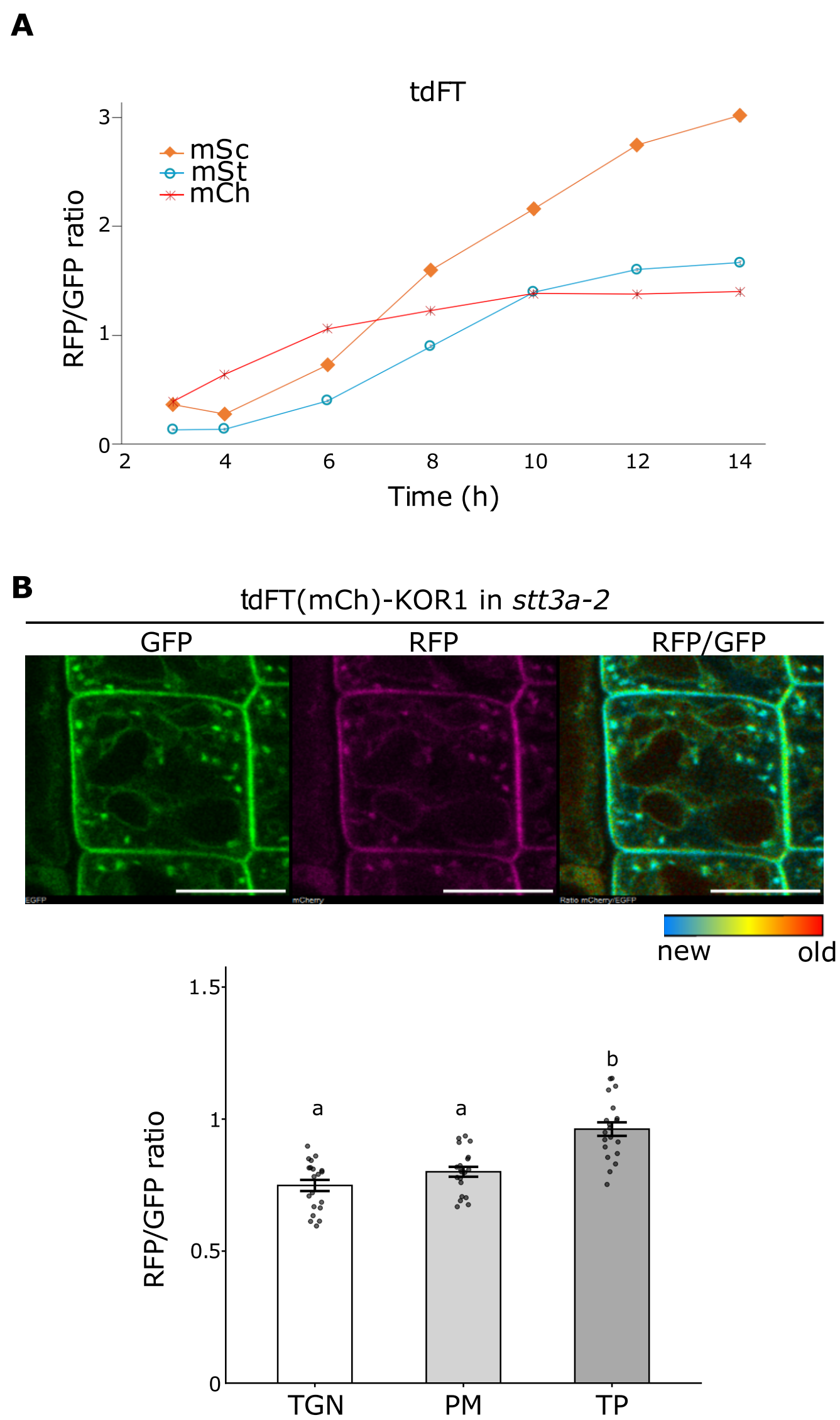

Fig. S2. Comparison of mCherry-based tdFT with selected tdFTs.

(A) Maturation kinetics of inducible cytosolic targeting tdFT with mCherry, mScarlet and mStrawberry. RFP/GFP ratio of cytosolic tdFT. *n* = 25. (B) Ratiometric values of various *KOR1pro*::tdFT(mCh)-KOR1 reporter at the *trans*-Golgi Network (TGN), plasma membrane (PM), and tonoplast (TP). Cellular RFP/GFP ratiometric values were observed in *stt3a-2* root tip cells and quantified. Scale bar = 10 µm. Error bars represent the standard error of the mean (SEM), *n* = 25. Different letters indicate significant differences ($p<0.05$) between genotypes (one-way ANOVA followed by Tukey's honestly significant difference post-hoc test).

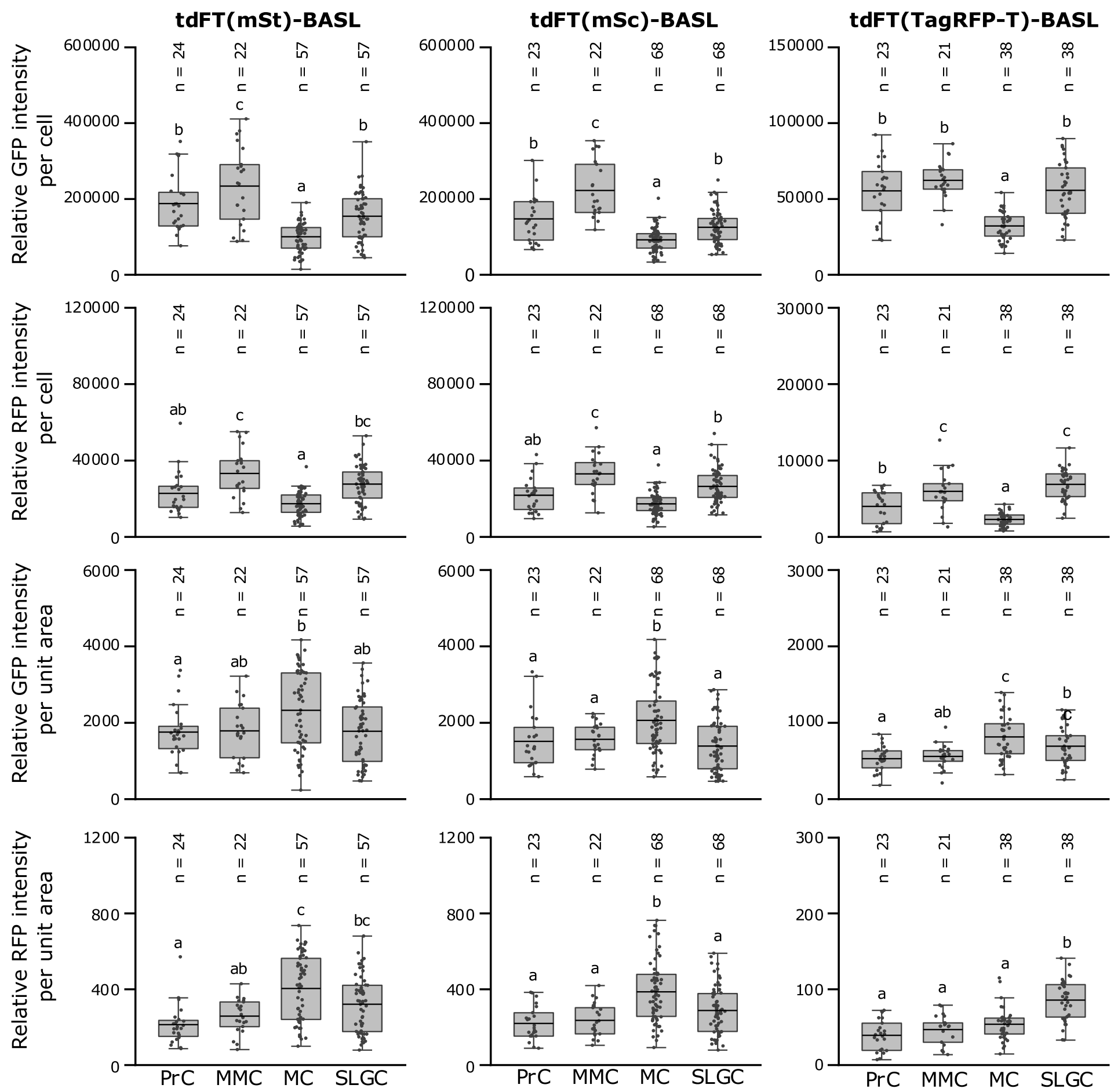

Fig. S3. Statistical analysis of relative GFP and RFP intensities derived from tdFT-BASL marker lines in different stomatal lineage cell types.

Fluorescence intensity values were collected from confocal images showing the expression of *BASLpro*::tdFT(mSt)-BASL, *BASLpro*::tdFT(TagRFP-T)-BASL, and *BASLpro*::tdFT(mSc)-BASL in stomatal lineage cells. Individual cell types were identified based on their morphology in the stomatal lineage. Prc, protodermal cells; MMC, meristemoid mother cells; MC, meristemoid cell; SLGC, stomatal lineage ground cell. Individual cell sizes and mean fluorescence intensities were measured (top two rows) and normalized to unit area (bottom two rows) using ImageJ. Error bars indicated mean ± SD (n, number of cells analyzed for each cell type). Different letters indicate significant differences ($p<0.05$) between genotypes (one-way ANOVA followed by Tukey's honestly significant difference post-hoc test).

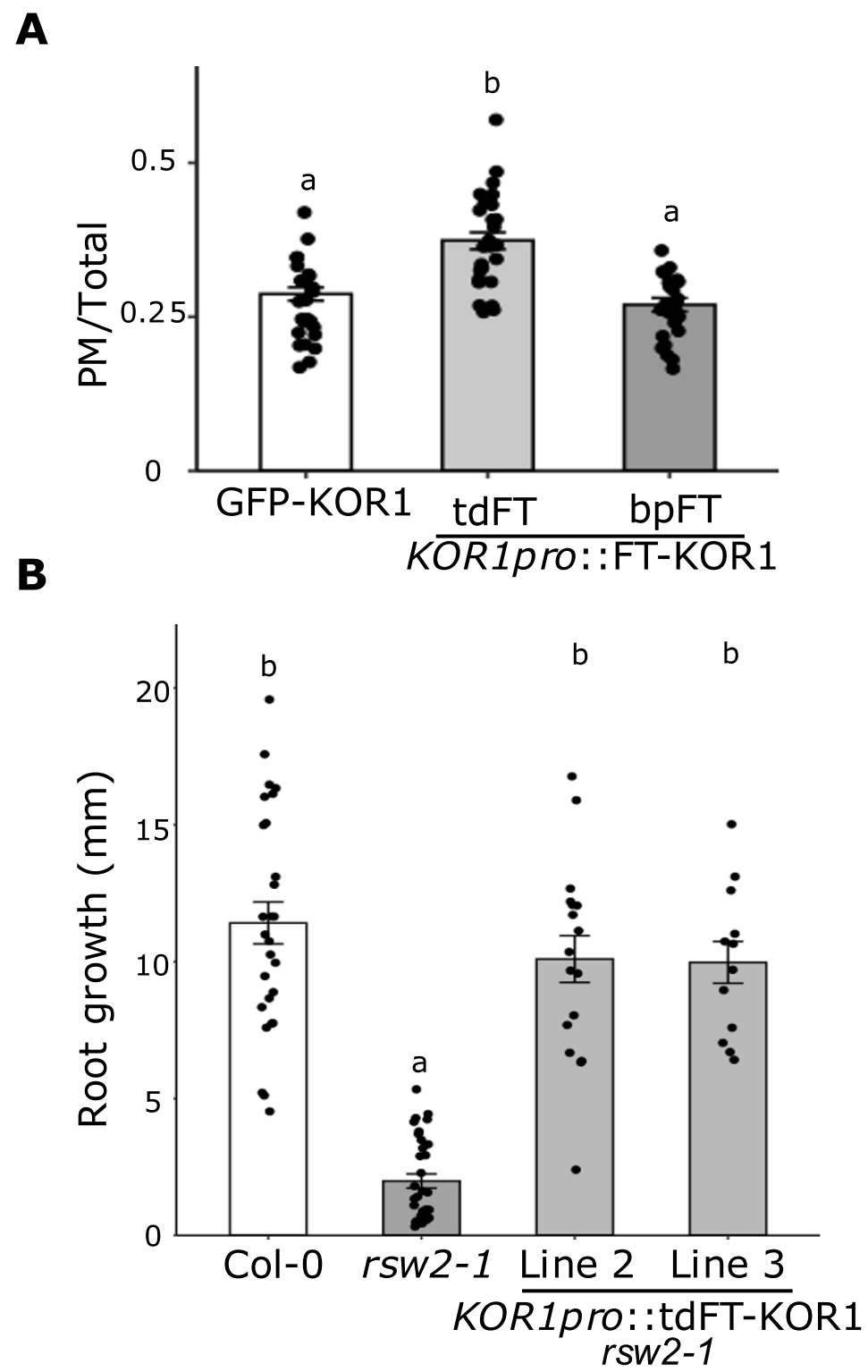

Fig. S4. Characterization of *KOR1pro*::tdFT(mSt)-KOR1.
(A) Relative GFP signal at the PM to the whole cell in GFP-KOR1 and *KOR1pro*::tdFT (mSt) and bpFT(mSt)-KOR1 in *stt3a-2* background. GFP signal intensity at the PM was divided by the total signal of cells. $n = 25$. (B) *KOR1pro*::tdFT(mSt)-KOR1 transgene rescue root growth of the *rsw2-1* host. Error bars represent the standard error of the mean (SEM), $n = 16$-31. Different letters indicate significant differences ($p<0.05$) between genotypes (one-way ANOVA followed by Tukey's honestly significant difference post-hoc test).

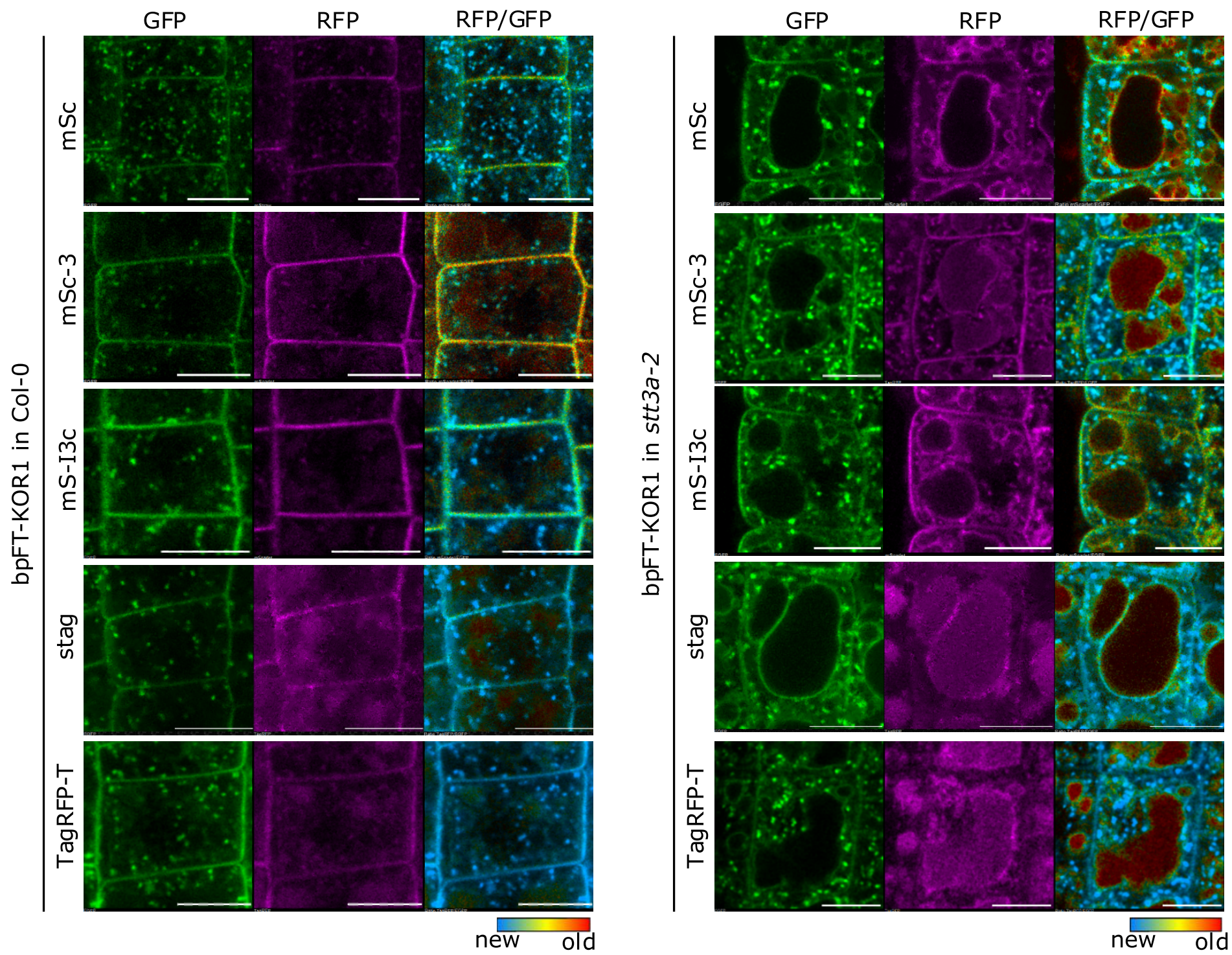

Fig. S5. Representative micrographs of the bpFT-KOR1 series used in the Fig. 8.
The root tip cells of three-day-old seedlings were used for observation. Scale bar = 10 µm.

Data S1. Plasmid list, targeting sequences, and primer list, and individual data values used in this study.

Sheet 1 contains an index of the supplementary datasets. Sheet 2 contains the plasmids used and generated in this study, including construct descriptions, GenBank accession numbers, and ABRC stock identifiers. Sheet 3 contains organelle-targeting sequences used for tdFT localization studies in Fig. 3. Sheet 4 contains oligonucleotide primer sequences used for cloning, mutagenesis, and construct preparation. Sheets 5–13 contain individual data values underlying figure panels with sample sizes <20, used for statistical analysis and generation of the corresponding graphs.
